# Roles of DNA Methylation in Cold Priming in Tartary Buckwheat

**DOI:** 10.3389/fpls.2020.608540

**Published:** 2020-12-07

**Authors:** Yuan Song, Zhifeng Jia, Yukang Hou, Xiang Ma, Lizhen Li, Xing Jin, Lizhe An

**Affiliations:** ^1^ Ministry of Education Key Laboratory of Cell Activities and Stress Adaptations, School of Life Sciences, Lanzhou University, Lanzhou, China; ^2^ Qinghai Academy of Animal Science and Veterinary Medicine, Qinghai University, Xining, China

**Keywords:** DNA metylation, cold response, agronomic trait, epigenetic regulation, Tartary buckwheat, cold priming

## Abstract

Plants experience a wide array of environmental stimuli, some of which are frequent occurrences of cold weather, which have priming effects on agricultural production and agronomic traits. DNA methylation may act as an epigenetic regulator for the cold response of Tartary buckwheat (*Fagopyrum tataricum*). Combined with long-term field observation and laboratory experiments, comparative phenome, methylome, and transcriptome analyses were performed to investigate the potential epigenetic contributions for the cold priming of Tartary buckwheat variety Dingku1. Tartary buckwheat cv. Dingku1 exhibited low-temperature resistance. Single-base resolution maps of the DNA methylome were generated, and a global loss of DNA methylation was observed during cold responding in Dingku1. These sites with differential methylation levels were predominant in the intergenic regions. Several hundred genes had different DNA methylation patterns and expressions in different cold treatments (cold memory and cold shock), such as *CuAO*, *RPB1*, and *DHE1*. The application of a DNA methylation inhibitor caused a change of the free lysine content, suggesting that DNA methylation can affect metabolite accumulation for Tartary buckwheat cold responses. The results of the present study suggest important roles of DNA methylation in regulating cold response and forming agronomic traits in Tartary buckwheat.

## Introduction

Plants can precisely perceive hypothermia through epigenetic regulation with short-term cold stress responses (Jung et al., 2013) or prolonged cold temperature changes (Luo and He, 2020). DNA methylation can function as an epigenetic regulator to potentially provide flexible genomic parameters for plants to respond to various cold stresses (Chinnusamy and Zhu, 2009; Huff and Zilberman, 2012; Sahu et al., 2013; Thiebaut et al., 2019). The stressful experiences of plants can change how they subsequently respond so that they have stronger stress tolerance when encountering sudden environmental changes in the future. In higher plants, this is known as “stress memory” or “stress imprinting” (Bruce et al., 2007), and the expression of stress memory genes is modulated by epigenetic mechanisms (D’Urso and Brickner, 2014; Chang et al., 2020). There have been many studies of stress memory, but the original report conducted by Zuther et al. (2019) proposed that cold memory can improve plant freezing tolerance by changes in gene expression and lipid and metabolite composition and defined the memory of cold acclimation as cold priming. Subsequently, there have been several reports of the involvement of epigenetics in plant stress memory (Ding et al., 2012; Yang et al., 2020b). However, the detailed mechanism of how epigenetic memory is involved in frozen memory has not yet been described.

Tartary buckwheat (*Fagopyrum tataricum*) is a pseudocereal that belongs to the genus *Fagopyrum* within the Polygonaceae family. Tartary buckwheat is strongly adapted to growth in adverse environments (such as harsh climates and nutrient-poor soils; Zhang et al., 2017, 2018; Zhou et al., 2017b). Additionally, Tartary buckwheat is a short-generation and a diploid, with highly enriched flavonoid products, facilitating its use as a potential model species to study low-temperature adaptability in plants. Recent studies in Tartary buckwheat have focused on the functional analysis of individual genes and developmental traits (Liu et al., 2018a,b, 2019b,c; He et al., 2019; Yao et al., 2019), but there have been no reports of global epigenetic regulation.

Low temperature or repeated diurnal temperature difference promotes the accumulation of flavonoids, mainly because low temperature greatly increases the activities of enzymes in the flavonoid synthesis pathway (Christie et al., 1994; Leyva et al., 1995; Caldwell et al., 2005), suggesting that cold-induced transcriptional events can lead to desirable agronomic traits in plants. Metabolites can also protect plant cell viability during adversity. Epigenetic information in “cis-memory” is stored as a state of local chromatin (e.g., by DNA methylation or histone modification) and “trans-memory” exists as movable factors (e.g., transcriptional repressors; Dean, 2017). Some studies have confirmed that epigenetic memory formed under stress may allow for the quick adaptation of plants to ambient temperature changes. The phenotypic variation that can be induced by epigenetic memory – but not hereditary variation – is important to cope with rapid changes in the environment (Latzel et al., 2016). Few details are known of how epigenetic memory allows for a response to a changing environment, and Tartary buckwheat is an ideal plant to investigate the related mechanisms.

In this study, long-term field testing and laboratory experiments revealed that Tartary buckwheat cv. Dingku1 presents frost resistance and other agronomic traits, such as higher germination rate, higher water content of seeds, and higher flavonoid content. Experiments were designed as different cold treatments, including cold memory (cold priming) and cold shock, and phenome, methylome, and transcriptome analyses were performed to investigate the frost resistance characteristics of Dingku1. The single-base resolution DNA methylomes of Dingku1 were characterized under different cold treatments and revealed the global loss of DNA methylation, with some locally hypermethylated sites. The Kyoto Encyclopedia of Genes and Genomes (KEGG) enrichment analysis revealed that DNA methylation significantly impacted the pathways of lysine degradation; pyrimidine metabolism; and the synthesis of isoquinoline alkaloids, metabolism-related genes (i.e., *FtDHE1*, *FtRPB*, and *FtCuAO*), and metabolites (i.e., lysine) to precisely regulate frozen memory. Treatment with a DNA methylation inhibitor interfered with the lysine level during the cold response, indicating that DNA methylation is critical for proper stress responses in Tartary buckwheat. These findings provide comprehensive insights into the development of cold priming and suggest guidelines for future breeding efforts in Tartary buckwheat.

## Materials and Methods

### Field Experimental Site

Field experiments were conducted at the China Oat and Buckwheat Industrial Technology System Haidong Comprehensive Test Station. Tartary buckwheat varieties that were recently bred to be frost tolerant were used.

**Figure 1:**
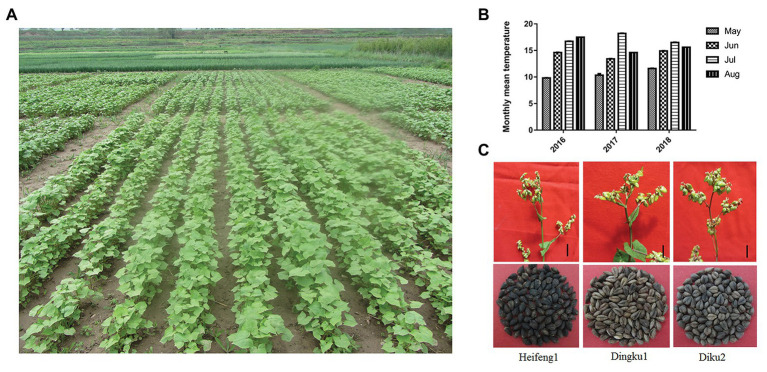
Tartary buckwheat and experimental site climate. **(A)** Tartary buckwheat Haidong experimental site in the light raining day, average altitude: 2,620 m. **(B)** Average temperature of Tartary buckwheat Haidong experimental site in recent 3 years of growing season (°C). **(C)** Collected flower branch and seed specimens for experimental measurement. Scale bar: 7 cm.

The core test base of the Haidong Comprehensive Test Station is located in Huangzhong, Qinghai Province; this site has a flat terrain and a cold and humid climate (Figure 1). The soil organic matter content was 98 g/kg, the content of the available N was 24.2 mg/kg, the available P (P_2_O_5_) was 13.43 mg/kg, the available potassium (K_2_O) was 110.32 mg/kg, and the pH was 8.4 (2016–2019 data). Meteorological data were provided by the Huangzhong Meteorological Bureau. Agronomic parameters [average yield per mu, plant height, length of tillering, thousand kernel weight (TKW), and grain yield] were measured as described (Liu et al., 2019a). Tartary buckwheat seeds were collected after maturity, and total flavonoids, total protein, total starch, and crude fat were determined according to national standards (GB5009.5-2016) and technical documents of the Standardization of Shenzhen City (SZDB/Z 349-2019).

### Plant Materials and Cold Treatments in Laboratory Experiments

After 4–8 h of soaking the seeds in ddH_2_O, the seeds were disinfected using a 15% NaClO solution and then placed in a culture dish with two layers of gauze. The culture dish was moved to a greenhouse and cultured until germination. Three-week-old seedlings were treated with 5-aza-2'-deoxycytidine (aza-dC, Sigma) that was added to the liquid nutrient medium (7 g/ml). Seedlings were divided into two groups in cold stress experiments. The first group was to test the changes in plant morphology, plant water content, and plant temperature before and after cold stress. The experiment was set at 4°C for 24 h, followed by recovery at room temperature (21°C) for 1 day. The second group was designed to simulate the field climate and was used to assess the character of different varieties. The experiment included plants subjected to cold memory (4°C for 6 h, then at room temperature for 18 h, repeated four times, and then placed at 0°C for 6 h), cold stock (not acclimated, directly exposed to 0°C for 6 h), and control groups with normal growth conditions.

### High Throughput Phenotypic Tests in Laboratory Experiments

Indoor high-throughput phenotypic observations were performed using image processing based on visible light (morphological test), infrared light (relative temperature measurement), and near-infrared light (relative water content test). A commercial phenotyping system (Scanalyzer3D, LemnaTec GmbH, Würselen, Germany) was used for image acquisition. Images of each plant were taken from the top and side views. Images were acquired and data preprocessing was organized into LemnaBase, a central database interface for the phenotyping system (Guo et al., 2017). Imaging, data acquisition, and data analysis of seeds and plants before and after cold treatment were carried out. Indicators of area, circumference, expansion degree, density, area of external polygons, circumferential length of external polygons, vertical length, horizontal length, and minimum diameter were performed to determine phenotypic differences in detail (An et al., 2017; Zhou et al., 2017a).

The chlorophyll values of plants were determined (Polyphenol-Chlorophyll Meter-Dualex Scientific+, Force-A, France) using the second and third fully expanded leaf near the plant center. Three indexes were measured: chlorophyll absorptivity (chlorophyll, Chl), anthocyanidin, and NBI (Cerovic et al., 2012). The one-way variance was calculated for multiple comparisons using SPSS 19.0, GraphPad Prim7 software and Microsoft Excel 2016.

### The Anti-freezing Physiological Index

Electrolyte leakage tests were performed as previously described (Song et al., 2019). Leaves were transferred to 15 ml tubes and placed in a freezer (XT5201-D31-R40C, XuTemp, China). The plants were exposed to freezing temperatures ranging from 10 to −6°C, and leaves without damage were then immersed in 10 ml ultrapure water (Milli-Q Advantage) and placed on a shaker at 4°C for 2 h. Electrolyte leakage was calculated as the ratio before and after leaves were boiled *via* a conductivity meter (DDSJ-308, Leici, China; Rohde et al., 2004).

The free proline was determined according to the following method. Briefly, 0.25 g of the plant material was weighed and combined with 1.75 ml 3% sulfosalicylic acid in a test tube and incubated in a boiling water bath for 10 min before centrifugation at 5,000 rpm for 10 min. Next, 0.5 ml of water, 0.5 ml of glacial acetic acid, and 1 ml of 2.5% acid trione were added to 0.5 ml of the supernatant, and the solution was developed for 30 min in boiling water. The absorbance at the wavelength at 520 nm was measured after cooling. According to the standard curve, the average content of free proline in each gram dry weight sample was calculated (Levy, 1980; Torrecillas et al., 1984).

After freezing treatment, the seedlings were transferred to 4°C for 12 h in dark conditions, put into normal condition for recovery for 3–5 days, and the number of seedlings that generated new leaves was counted as the survival rate (Ding et al., 2019). Membrane oxidation in cold stress was assessed by measuring the MDA level (Wang et al., 2017).

### Whole-Genome Bisulfite Sequencing and Analysis

Total DNA was extracted using the QIAamp Fast DNA Tissue Kit (Qiagen, Dusseldorf, Germany) following the procedure given by the manufacturer. DNA samples were fragmented using sonication and subjected to bisulfite conversion and second-generation sequencing.

The Accel-NGS Methyl-Seq DNA Library Kit (Swift, MI, United States) was utilized to attach adapters to single-stranded DNA fragments for library construction. Paired-end, 2 × 150 bp sequencing was performed at the Hangzhou Lianchuan Biotechnology Center using an Illumina Hiseq 4,000 platform. Library construction, sequencing, and bioinformatics analysis are described in the SI Appendix, Supplementary Information Text 1.

### RNA Analysis

Total RNA was extracted with a Trizol reagent (Invitrogen, CA, United States) following the procedure provided by the manufacturer. Next, 1 μg of RNA and oligo dT primers were used to synthesize cDNA in a 20 μl reaction to create the final cDNA library using an mRNA sequence sample preparation kit (Illumina, San Diego, CA, United States). The average insert size for the paired-end libraries was 300 bp (±50 bp). Paired-end sequencing was done on an Illumina Hiseq4000 (LC Sciences, United States) following the vendor’s recommended protocol. Read mapping, transcript abundance estimation, and differential expression quantitation were performed as described in the SI Appendix, Supplementary Information Text 1.

### Real-Time Quantitative RT-PCR

Total RNA was extracted from the frozen tissue using Tiangen’s RNA prep pure plant kit (Cat.DP432, Beijing, China) according to the manufacturer’s instructions. Next, 2 μg RNA was used for the first strand of cDNA synthesis using reverse transcriptase (Thermo Scientific, #EP0441). Real-time PCR amplification was carried out with the Bio-Rad CFX96 system using SYBR Green I (Takara, DRR081A, Dalian, China). PCR conditions were 3 min at 95°C followed by 40 cycles of the following: 95°C for 10 s, 60°C for 15 s, and 72°C for 15 s. The primer pairs used were *FtCuAO* 5'-ACCTCAGGTGAAGCAGTCAA-3' and 5'-GGGATTTCGCACCCTCATTC-3'; *FtRPB1* 5'-CTCACGACAACCACCATTCC-3' and 5'-CCTCCTTGTGTGGAGTGTCT-3'; and *FtDHE1* 5'-CAGAGGAGCTTGCTTGGTTG-3' and 5'-CGCAAATGGCAGACACTGAT-3'. The *FtH3* gene was amplified as a reference gene, since its expression is unaffected by abiotic treatment (Li et al., 2019), using primers 5'-GAAATTCGCAAGTACCAGAAGAG-3' and 5'-CCAACAAGGTATGCCTCAGC-3'.

### Measurement of Rutin and Lysine Content

Fresh seedlings (1 g) of Dingku1 were frozen and ground in liquid nitrogen. The rutin content was analyzed by high-performance liquid chromatography (HPLC) from triplicate independent extractions as described previously (Zhang et al., 2017). Briefly, dried seedlings (100 mg) were ground, then mixed with 500 μl Na-S™ buffer (2% sodium citrate, 1% HCl, and 0.1% benzoic acid; Beckman, United States) for 30 min in a mixer, and extracted for 10 min *via* ultrasonication. The free lysine content was analyzed using an A200 Amino Acid Analyzer (Aminosys, Germany), from triplicate independent extractions as described previously (Yang et al., 2016).

## Results

### Evaluation of Traits of Tartary Buckwheat Varieties by Field-Scale Experiments

To screen for high-quality Tartary buckwheat resources adapted to the high altitude and cold climate of the Tibetan Plateau, dozens of Tartary buckwheat varieties were subjected to a long-term adaptive assessment at the Haidong comprehensive test station (Qinghai Academy of Animal Science and Veterinary Medicine) using advanced field real-time detection equipment and unmanned aerial vehicle (UAV) detection managements. Through long-term observation, three varieties were focused on for further field experimental tracking and indoor experiments: Heifeng1, Diku2, and Dingku1. The experimental site was established in Huangzhong, Qinghai Province (approximately 36°28'N, 101°37'E; Figure 1A), with an average altitude of 2,620 m, an annual average temperature of 3.7°C, and a growing season average temperature of 14°C (Figure 1B). Plants were observed extensively, such as flower branch and seeds (Figure 1C). Thereinto, Tartary buckwheat cv. Dingku1 exhibited traits of late flowering, higher germination rate, and higher content of total flavonoids and starch in seeds (Supplementary Figures S1, S2).

### Evaluation of Frost Resistance in Tartary Buckwheat Varieties by Laboratory Bench-Scale Experiments

To confirm the findings of the field data, a large-scale and accurate phenotypic analysis of three plant varieties – Heifeng1, Diku2, and Dingku1 – was carried out under laboratory control conditions (Figure 1B; Supplementary Figure S2). The morphological characteristics of seeds were observed within the 3 days before and after germination. The results showed that there was no significant difference in the morphology of the seeds from the three varieties during germination, but the seeds from Dingku1 were of slightly smaller volume than those of the other two varieties before and after germination, and the compact density was relatively larger (Figure 1B; Supplementary Figures S2a, S3). During germination, the seed temperature and seed water content of Dingku1 were higher compared with seeds from the other two varieties (Supplementary Figures S1b,c, S4).

**Figure 2:**
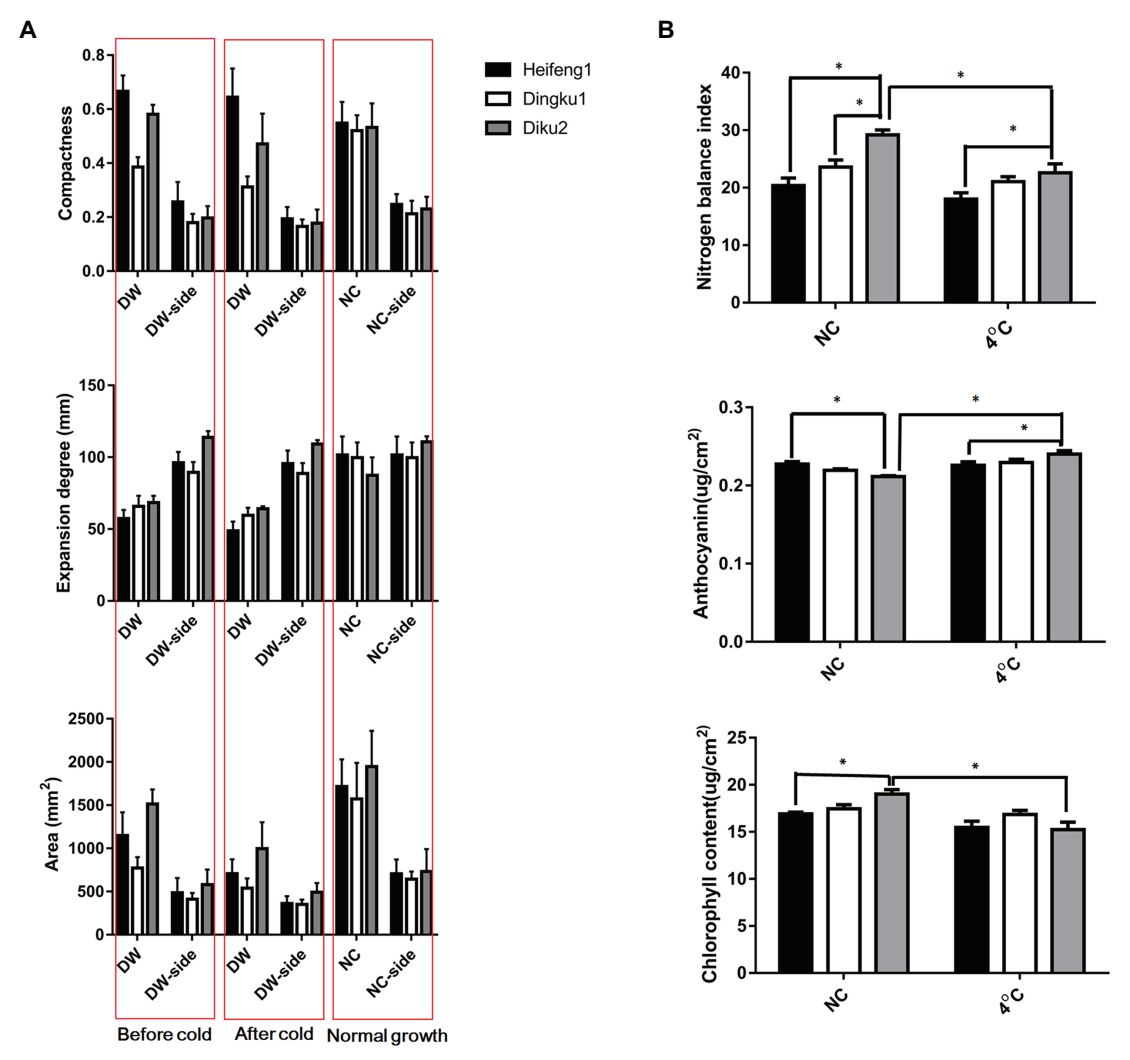
Phenotypic observation and physiological test in cold experiment of 3-week-old seedlings. **(A)** Morphological observation before and after 4°C treatment. DW, Low temperature treatment groups from the top surface testing; DW-side, Low temperature treatment groups from the side face testing; NC, non-specific control. **(B)** Physiological test between the control and cold treatments. The every mean value was from more than 30 independent plant measurements, and error bars indicated ± SD. Analysis was performed with one-way ANOVA followed by Tukey-Kramer *post hoc* analysis. ^*^ *p* < 0.05.

An experiment of cold treatment (4°C, 24 h) followed by 1 day of recovery was carried out, and the frost resistance was assessed by observing, in detail, the morphology of seedlings with three leaves by optical imaging and image analysis technology. Compared with the other two varieties, the seedlings of Dingku1 were shorter, narrower in width, and more compact (Figure 2A; Supplementary Figure S5). Dingku1, however, did not differ significantly between groups and within groups in anthocyanin and chlorophyll test, and differences were observed in the control group only in nitrogen balance index test (Figure 2B). The temperature and water content of seedlings were measured before and after cold treatment in three varieties. In contrast, Dingku1 had higher water content and plant temperature after cold treatment (Supplementary Figure S6).

**Figure 3:**
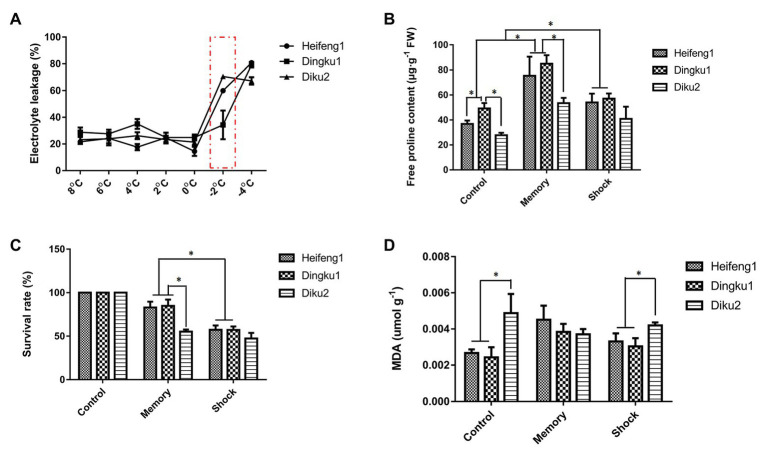
Physiological and biochemical tests in cold acclimation and freezing treatments of Tartary buckwheat three varieties. **(A)** Electronic leakage rate of Tartary buckwheat three varieties in low temperature. **(B)** Change of proline content in different low temperature treatments. **(C)** Survival rate in different low temperature treatment. **(D)** Malondialdehyde (MDA) content in different low temperature treatments. The mean value was from more than 30 independent plant measurements, and error bars indicated ± SD. Analysis was performed with one-way ANOVA followed by Tukey-Kramer *post hoc* analysis. ^*^ *p* < 0.05.

Next, experiments of cold acclimation (memory) and freezing stress (shock) were designed to simulate field weather. Through physiological and biochemical indexes, it was determined that Dingku1 exhibited characteristics for stronger frost resistance (Figure 3). Compared to the other varieties, Dingku1 exhibited a lower ion leakage rate (Figure 3A), higher free proline content (Figure 3B), higher survival rate (Figure 3C), and lower malondialdehyde (MDA) content (Figure 3D) during freezing stress. Therefore, Dingku1 was selected for additional study on the cold environmental adaptation mechanism.

In addition, the memory group (cold priming) with cold acclimation exhibited better performance (Figure 3), higher free proline content (Figure 3B), and higher survival rate (Figure 3C) after being subjected to the second freezing stress, compared to the control plants grown at 16–21°C or the plants that experienced freezing shock without prior cold acclimation.

### Tartary Buckwheat cv. Dingku1 DNA Methylomes During Cold Stress

DNA methylation is closely related to plant frost resistance (Song et al., 2015). To characterize the Tartary buckwheat Dingku1 methylome during cold stress, a whole-genome bisulfite sequencing was performed and single-base resolution maps of DNA methylation were generated for three test groups: the control group (normal temperature), cold memory group (priming: 4°C for 6 h, followed by 21°C for 18 h, repeated for 4 days, then 0°C for 6 h), and cold shock (0°C for 6 h directly, without priming).

Each methylome was sequenced to >10-fold coverage per strand, covering >52% of the genomic cytosine positions (Supplementary Table S1). For each sample, at least 200 M (C: 235 M; M: 218 M; S: 227 M) paired-end reads (read length = 150 bp) were produced. Approximately 75% (C: 74.96%; M:76.66%; and S:76.19%) of the reads were mapped to the reference genome using Bismark (Krueger and Andrews, 2011), covering >90% of the genome (C: 92.94%; M:92.13%; and S:92.57%; Supplementary Table S1).

**Figure 4:**
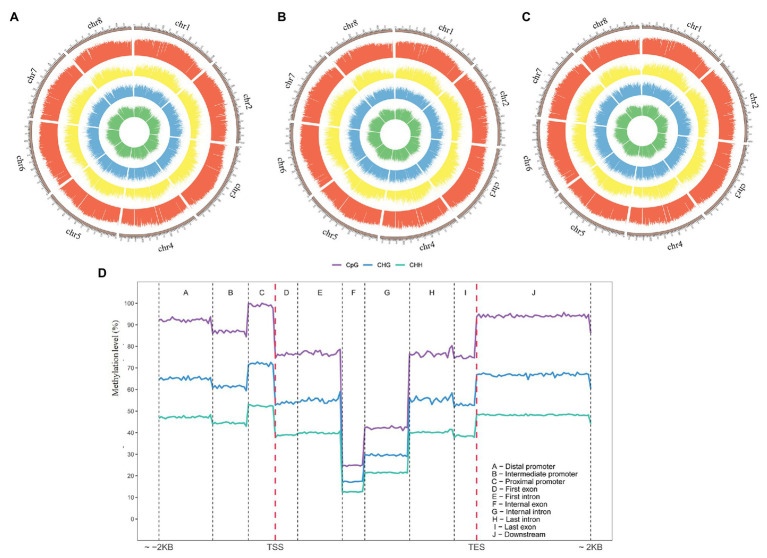
Characterization of Tartary buckwheat cv. Dingku1 methylomes. **(A–C)** The rings indicate (from outside to inside, were quantified per 1 Mb window) density plot of 5-methylcytosine in sequence contexts in different cold treatments. The methylation of CpG (red), CpHpG (yellow), CpHpH (blue), and total C levels (green) were mCG, mCHG, and mCHH, where mC signifies 5-methylcytosine. H = A, C or T. Chromosome name and scale are indicated on the outer rim. The level of methylation at the corresponding position is represented by the height of the column on the circle map. **(A)** The control group. **(B)** The cold memory group. **(C)** The cold shock group. **(D)** The different methylation (CG, CHG, and CHH) level of cytosine in featured regions of the genome.

Tartary buckwheat is the only Polygonaceae plant whose methylome has been reported to date. This analysis revealed the details of methylated base sites (Figure 4; Supplementary Table S2). The results show different 5-methylcytosine distributions in different regions for the control and the other two cold treatment groups, with most of the regions with decreasing in CpG methylation mapping to the VIII chromosome (Figures 4A–C). There are several functional genes with hypomethyl-modifications, such as the ubiquitin-activating enzyme E1, helicases, and hydrolases, with potential roles in the cold shock response that are regulated by DNA methylation (Supplementary Table S4). There was different methylation of CHH for all three treatments, with slightly higher methylation densities of CHH in the cold memory group compared to that in the other two treatments, as well as significantly lower methylation density in the cold shock group than that in the other treatments (Figures 4A–C).

An analysis of the distribution of DNA methylation in Tartary buckwheat showed the enrichment of DNA methylation in the proximal promoter region (5' terminal of TSS) and downstream region (3' terminal of TES; Figure 4D), indicating increased DNA methylation in the 5' and 3' flanking regions of genes. In addition, genes were characterized by a high enrichment of CG methylation and the moderate enrichment of CHG methylation and CHH methylation (Figure 4D), suggesting that CG was the primary type of cytosine methylation in Tartary buckwheat gene-transcription domains. Little difference was observed in the distribution of methylation among the three test groups (Supplementary Figure S7), and similar trends in methylation levels were observed for the three groups. In the control group, the total DNA methylation level was 24.62%, with 78% CG methylation, 40.28% CHG methylation, and 11.1% CHH methylation (Supplementary Table S3).

### DNA Methylation in Dingku1 Decreases During Cold Stress

**Figure 5:**
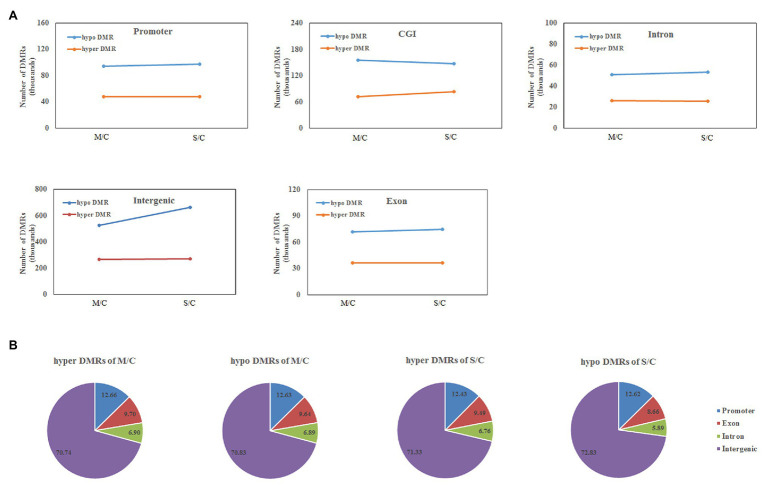
Genome-wide decrease in DNA methylation during Dingku1 cold responses. **(A)** Numbers of hyper- and hypo-DMRs in cold memory (M) and cold shock (S) relative to the control (C) in different genomic regions. **(B)** The pie chart shows the proportion of genomic component in hyper- and hypo-DMRs.

As shown in Supplementary Figure S8, the principal component analysis (PCA) revealed significant differences among the three groups. The average DNA methylation levels were calculated and a decrease in the global cytosine methylation level from the control group to the cold treatment group (from 23 to 24.5%) was found. Differentially methylated regions (DMRs) were identified and it was found that the change of DNA methylation was mostly due to a decrease in intergenic methylation (Figure 5A).

To characterize the change of DNA methylation for different treatments, a method based on Fisher’s exact test was used to identify DMRs between methylomes (Ausin et al., 2012). The methylome of the cold memory group or the cold shock group (M/C and S/C, respectively) was compared with that of the control group. As shown in Figure 5, both hyper-DMRs and hypo-DMRs were detected in similar proportion in M/C and S/C (Figure 5B), but there were more hypo-DMRs in all comparisons, with 526,103 intergenic hypo-DMRs identified in the M/C comparison and 664,615 in the S/C comparison, whereas only 267,055 hyper-DMRs were observed for M/C and 272,784 for S/C. These results suggest a global decrease in DNA methylation in cold treatments, especially cold shock. Hypo-methylation was mainly observed in intergenic contexts, although decreased methylation was also observed in the CGI, promoter, intron, and exon regions to a lesser extent, suggesting that they have an important regulatory function of intergenic regions in the Tartary buckwheat genome.

### Correlation Between DNA Methylation and Gene Expression in Cold Stress of Dingku1

Transcriptome profiles for Dingku1 were generated by RNA-seq analysis – with three biological replicates for seedlings under the same treatments used to assay methylation – to investigate whether the observed decrease in DNA methylation during a cold response was associated with changes in gene expression. A total of 31,391 genes and 34,067 transcripts were included in the analysis, with more upregulated genes and transcripts than downregulated genes and transcripts in the two treatment groups relative to the control group (Supplementary Figure S9). Furthermore, 24,931 differentially expressed genes (DEGs) were identified, of which 1,893 and 1748 were significantly differentially expressed in the cold memory group and the cold shock group, respectively, relative to the control group.

Considering the association between DNA methylation and gene expression, hyper- and hypo-DMR-associated genes were analyzed during cold treatments. There were 377,514 and 382,440 hyper-DMR in the cold memory and the cold shock groups, respectively, which were significantly lower than the numbers of hypo-DMRs for the two groups. There were 742,756 and 889,778 hypo-DMRs in cold memory and cold shock, respectively.

Statistical analysis revealed the potential coupling of the changes in DNA methylation and gene expression. A total of 21,335 genes were identified as DMR-associated genes in the buckwheat genome (FPKM >1). Among these genes, 336 upregulated and 217 downregulated DEGs were identified as hypo-DMR-associated genes, and 143 downregulated and 189 upregulated DEGs were identified as hyper-DMR-associated genes in the cold memory compared to the control. Additionally, 341 upregulated and 205 downregulated DEGs were found to be hypo-DMR-associated genes, and 125 downregulated and 198 upregulated DEGs were determined to be hyper-DMR-associated genes in the cold shock vs. the control (Supplementary Table S5). As was found previously for tomato and orange fruit ripening (Lang et al., 2017; Huang et al., 2019a), DNA hypermethylation is associated with gene activation in Tartary buckwheat cold response. The results of the present study suggest that DNA methylation may play a positive role in regulating the Tartary buckwheat response when exposed to external stresses. In the cold treatments relative to the control (M/C and S/C), there were four clusters: up-up (upregulated DEGs with hyper-DMR), up-down (downregulated DEGs with hyper-DMR), down-up (upregulated DEGs with hypo-DMR), and down-down (downregulated DEGs with hypo-DMR).

Gene Ontology (GO) analysis was performed to understand the potential role of DNA methylation in Tartary buckwheat cold tolerance. With an overall trend of hypomethylation during cold treatment, the analysis was focused on down-up genes (DNA methylation decreased and the expression increased) in M/C and S/C, which were annotated as members of 203 and 199 terms, and the significantly enriched GO terms were 29 and 38 (*p* < 0.05), respectively. The most significantly enriched genes in M/C are related to integral components of membranes, suggesting that membrane structure is related to the formation of frozen memory, but these genes were not significantly expressed during cold shock (Supplementary Figure S10a). GO analysis revealed 30 ATP binding-related genes enriched in the S/C down-up cluster, suggesting that the activation of these genes occurs by cold shock induction of DNA hypomethylation (Supplementary Figure S10b). ATP binding is an important physiological activity in plants. For example, ATP binding to Cryptochrome2 (cry2) and some other plant cryptochromes promotes the activation of light receptors and increases stress resistance (Eckel et al., 2018).

The same enrichment of GO terms was observed in different freezing treatments (richness factor > 0.8), including genes related to protein phosphorylation, DNA repair, endosome organization, endoplasmic reticulum organization, sulfuric ester hydrolase activity, D-alanine ligase activity, and mitotic cell cycle, suggesting that these tissue and cell activities were involved in the cold response and tolerance of plants. There were also obvious differences in the enrichment of GO terms for the different freezing treatments. For example, in the upregulated DEGs with hypo-DMR (down-up) for cold memory treatment relative to the control (M/C), dephospho-CoA kinase activity, voltage-gated calcium channel activity, and cell adhesion were significantly enriched (Supplementary Figure S10a). For cold shock treatment relative to the control (S/C), glycolipid biosynthetic process, regulation of cell shape, and cell division were specifically enriched (Supplementary Figure S10b). The enriched GO terms were compared for the two different freezing treatments (M/S). The results showed that the number of enriched genes was more different (>80%) for the metabolic process, oxidation-reduction process, and ribonucleoprotein complex, as well as the ribosome, nucleolus, intracellular, and protein binding in M/S (Supplementary Figure S10c). Differential enrichment was observed for some enzymes (including racemase, epimerase, and catechol oxidase) and some metabolic processes (including glycerol metabolism, cellular carbohydrate metabolism, and malate metabolism; Supplementary Figure S10d), indicating differences in DNA replication, RNA transcription, and the tricarboxylic acid cycle due to different freezing treatments inducing different response mechanisms by demethylation regulation.

The effect of cold memory on the adaptability of Tartary buckwheat to a cold environment wanted to be better understood, and the KEGG analysis revealed the enrichment of genes in plant metabolic pathways in comparison of different treatment groups (Supplementary Figures S11a–c). Glycosylphosphatidylinositol (GPI)-anchor biosynthesis and D-Alanine metabolism showed a significant increase in the cold memory group (Supplementary Figure S11a). Plant GPI-anchored protein controls the cellulose content of the cell wall and guides the orientation for cell expansion (Cao et al., 2012). Frozen memory appears to activate the expression of this kind of protein to improve the freezing resistance of plants. Genes related to D-glutamine and D-glutamate metabolism, pyruvate metabolism, methane metabolism, biotin metabolism, and lipoic acid metabolism were significantly increased in the M/S cluster (Supplementary Figure S11c); however, little is known about the role of these pathways in countering freeze stress. There was an obvious difference between plants subjected to the slow accumulation of freezing signals and the rapid response when subjected to freezing.

**Figure 6:**
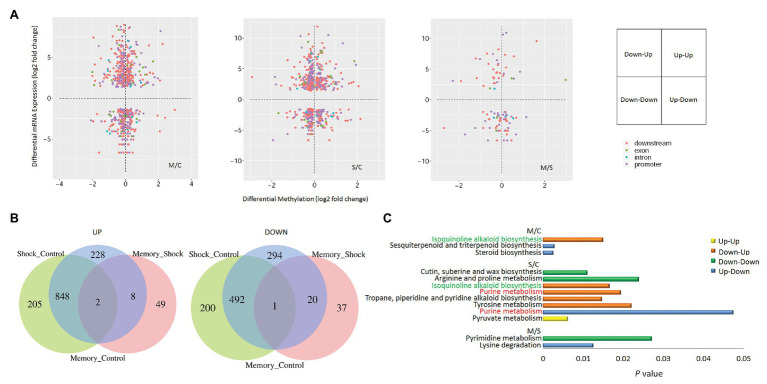
Correlation between DNA methylation and gene expression levels during Dingku1 cold responses. **(A)** The two-dimensional scatter diagram of correlation analysis of differential gene parts and DNA methylation enrichments. The x-axis represents DNA methylation levels of gene promoter region 2 kbp (purple dots), downstream −2 kbp (pink dots), exon (green dots), and intron (blue dots). The y-axis represents expression levels of corresponding genes. **(B)** Indicated Venn diagrams of the expressed genes drew by comparative analysis of significant differential expressed genes (DEGs) with DMRs. **(C)** Significant different KEGG pathways of DEGs were illustrated (*p* < 0.05). Hypomethylated up-DEGs (down-up), hypomethylated down-DEGs (down-down), hypermethylated up-DEGs (up-up), and hypermethylated down-DEGs (down-down) are shown respectively.

**Figure 7:**
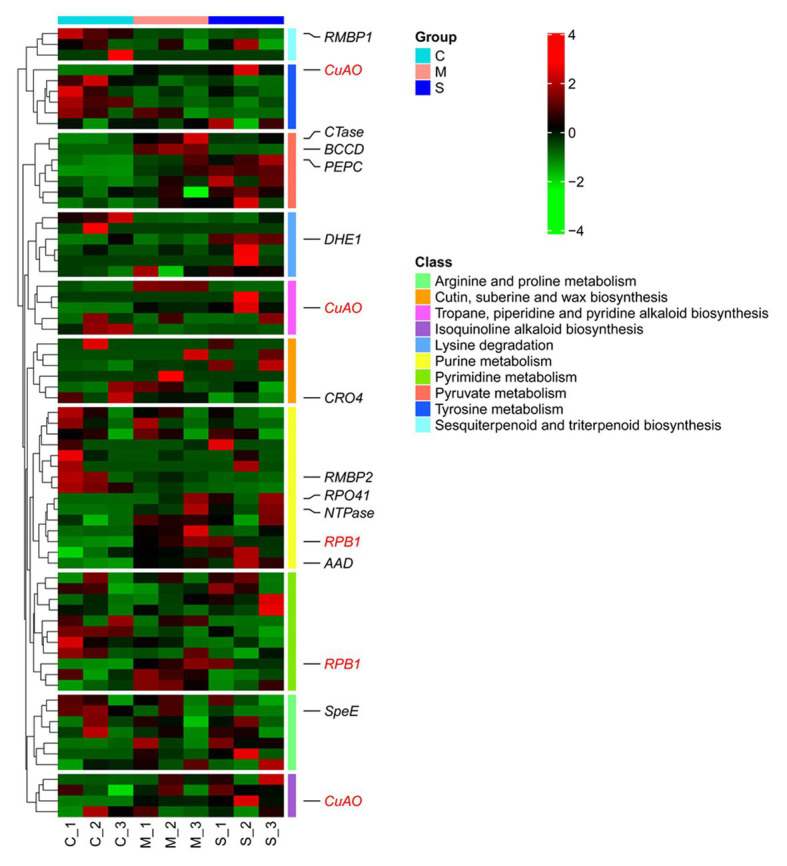
Heatmap of differential gene expression among various treatments. Genes with an adjusted *p* < 0.05 and relative fold change|log2FC| > 0.5 are displayed. The genes marked red are involved in multiple pathways.

A two-dimensional scatter diagram of correlation analysis was constructed to illustrate the overall relationship between differentially expressed genes and DNA methylation (Figure 6A; Supplementary Table S7). As shown in the Venn diagram, there is an overlap of the DEGs identified in the different treatments (Figure 6B). The numbers of down-regulated genes specifically expressed in each treatment were 294 (M/C), 200 (S/C), and 37 (M/S), and the numbers of upregulated genes were 228 (M/C), 205 (S/C), and 49 (M/S). Heatmap analysis showed a significant downregulation of transcription factors MYB108, Gcn5-related N-acetyltransferase (GNAT), and the late elongated hypocotyl (LHY), as well as a significant upregulation of WRKY40, BHLH, and JMJ30 in M/C. LHY, WAXY, and CYP were downregulated, and ERF053, WRKY40, and JMJ30 were upregulated in S/C (Supplementary Figure S12; Supplementary Table S6). The results showed shared and distinct aspects of the two cold coping strategies. The significantly different KEGG pathways were combined with metabolomics analysis (Figure 6C). The important genes in the pathways were demonstrated (Figure 7). There was a drop in methylation levels and an increase in the transcriptional level of copper amine oxidase (*CuAO*), which is a hypo-DMR-associated gene in cold treatment. This gene is involved in multiple pathways, such as the isoquinoline alkaloid biosynthesis pathway in M/C, as well as in the tropane, piperidine, and pyridine alkaloid biosynthesis pathway, and tyrosine metabolism pathway in S/C. RNA polymerase II’s largest subunit (*RPB1*) and dehydrogenase E1 component (*DHE1*) showed differences in DNA methylation enrichment and transcriptional level in different cold treatments (M/S). The analysis of the present study suggests that DNA methylation can mediate the Tartary buckwheat cold response through the regulation of cold-induced genes.

### Change in DNA Methylation Level Affects the Expression of Different Genes and Metabolites

**Figure 8:**
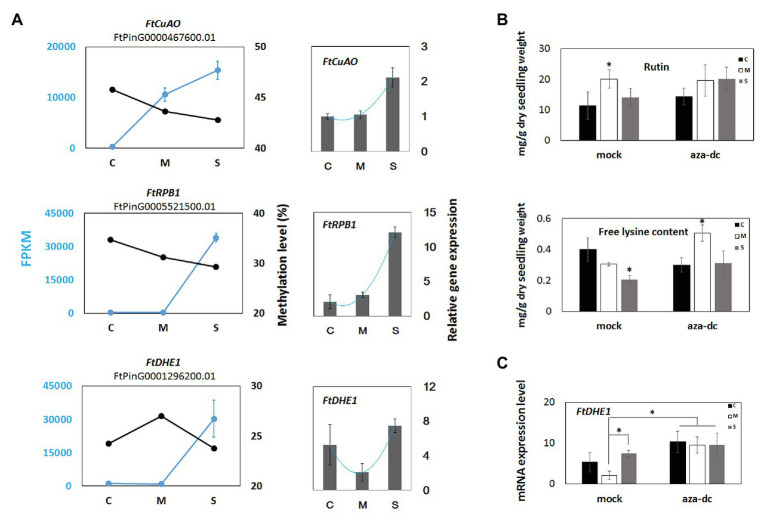
The significance of DNA methylation for Tartary buckwheat cold responding. **(A)** DNA methylation and expression levels of cold induced differential genes during Dingku1 cold responses. Blue lines with filled dots represent transcript abundance. Black lines with filled dots represent methylation level gene promoter region (2 kb). Histograms represent qRT-PCR test results. Data shown are mean ± SE (n = 3). **(B)** Rutin and lysine tests. Dingku1 were treated with DNA methylation inhibitor, 5-aza-2'-deoxycytidine (aza-dc), or mock (ddH2O) in different treatments, the control group (C), cold memory group (M), cold shock group (S). ^*^ *p* < 0.05, error bars indicate means ± SE (n ≥ 12). **(C)** mRNA of *FtDHE1* expression levels in aza-dc treatment. Data shown are mean ± SE (n = 3). Analysis was performed with one-way ANOVA followed by Tukey-Kramer post hoc analysis. ^*^ *p* < 0.05.

The correlation between promoter methylation levels and transcript levels of genes related to lysine, pyrimidine, alkaloid, and flavonoid metabolism were further examined. Promoter methylation levels and gene expression levels were determined for individual genes for the two cold treatments. For most genes, the expression levels and promoter methylation levels exhibited a negative correlation (Figure 8A). For isoquinoline alkaloid biosynthesis, pyrimidine metabolism, and lysine degradation, the expression levels of *FtCuAO*, *FtRPB1*, and *FtDHE1* showed increased trends, while their promoter cytosine methylation levels were decreased in cold treatments (Figure 8A).

The transcript abundances were measured for three selected unigenes by qRT-PCR to validate transcriptome data. Consistent cold responses were detected between the qRT-PCR analysis and RNA-Seq data (Figure 8A). *CuAO* acts in the isoquinoline alkaloid biosynthesis pathway, relating to H_2_O_2_ production with polyamine catabolism (Rea et al., 2004) and ABA-induced stomatal closure (Fraudentali et al., 2019). *CuAO* catabolizes polyamines and is associated with stress responses (Rea et al., 2004; Fraudentali et al., 2019). *FtCuAO* showed a significant expression increase in the cold shock group by qRT-PCR, which was consistent with the observed response detected by RNA-Seq. The *FtRPB1* gene is involved in purine and pyrimidine metabolism and exhibited a significant increase by qRT-PCR in the cold shock group. *FtDHE1* participates in lysine degradation and showed a significant decrease in the memory group but a significant increase in the cold shock group based on the qRT-PCR analysis. These genes also exhibited demethylation (Figure 8A).

To further analyze the significance of DNA methylation for metabolic pathways, the DNA methylation inhibitor 5-aza-2'-deoxycytidine (aza-dc) was added into the Dingku1 nutrient solution. Rutin and lysine levels were detected in plants grown in this solution. The results showed that aza-dc had little effect on rutin accumulation but had a great influence on lysine level, especially in the cold memory group (Figure 8B), and we further examined the expression of *FtDHE1* under aza-dc treatment, the results also showed that *FtDHE1* expression has been disturbed (Figure 8C), suggesting a role of DNA methylation in lysine synthesis and metabolism pathway in cold priming.

## Discussion

Temperature is a key factor affecting growth and development in plants. However, the temperature fluctuates under natural conditions, both daily and seasonally. Different plants have evolved a variety of mechanisms to sense complex and variable temperature signals and to regulate their growth, development, or behavior to adapt to changes in environmental temperature. Plants use complex mechanisms to grow under natural conditions and perceive changes of ambient temperature and store temperature memories to better adapt and form desired agronomic traits. In this study, Dingku1 (a Tartary buckwheat variety) was identified as having a high flavonoid content and good temperature tolerance (Figures 2, 3). Repeated cold simulation (cold priming) was used together with high-throughput measurement to find patterns of DNA methylation and relationships between genes and metabolites. The results showed a significant involvement of DNA methylation with enrichment changes of genes and metabolites related to isoquinoline alkaloid biosynthesis, lysine degradation, and pyrimidine metabolism (Figures 6–8).

Environmental temperatures affect virtually all aspects of plant growth and developmental processes. Plant cells can create various chromatin states for stress-responsive gene expression that are required for the adaptation to harsh environmental conditions (Zhao et al., 2020). The epigenetic marks are deemed as environment-dependent patterns through calculations using large populations (Alonso et al., 2019). Environmental factors modulate the epigenomic landscape and regulate adaptive responses in plants. Cytosine methylation (5-mC) is an epigenetic mark associated with developmental programs and stress responses and maintains genome stability by preventing mobilization of transposable elements (TE; Law and Jacobsen, 2010). Environmental cold temperatures can induce the transcription of transposons and lead to desirable agronomic traits in plants (Butelli et al., 2012). The 5-mC profile of a locus reflects the balance between pathways allowing accurate maintenance or a change in DNA methylation, with dynamics affected by abiotic and biotic stresses as well as developmental programs to result in phenotypic changes. This is seen by the overall change of the DNA methylation level during ripening of orange (Huang et al., 2019a), tomato (Zhong et al., 2013; Gallusci et al., 2016; Lang et al., 2017), and strawberry fruit (Cheng et al., 2018). DNA methylation may also be involved in the heterotic traits in broccoli (Li et al., 2018). These observations suggest the potential to alter the methylome to alter adaptation in plants and promote breeding (Molinier, 2020).

The results of the present study show a global loss of DNA methylation during cold treatments, with significant changes in chromosome VIII in cold shock (Figure 4C). No similar results were reported, and this result will continue to be validated in the future. A detailed analysis of regions in S/C showing differential expression was conducted and DEGs were classified by GO and KEGG analysis. Terms involved in the biological process were enriched, such as the “carbohydrate metabolic process,” “transport,” “proteolysis,” “oxidation-reduction process and lipid metabolic process,” “metal ion binding,” and “amino acid metabolism,” suggesting DEGs regulated by methylation are closely related to the metabolic activity (Supplementary Figure S13).

Prior studies have noted the relationship between epigenetic mechanisms and environmental changes, such as sulfur homeostasis by DNA and histone methylation (Huang et al., 2019b); salicylic acid metabolism in heterosis regulated by decreased DNA methylation 1 (DDM1; Zhang et al., 2016); indole-3-acetic acid (IAA) metabolism and transport regulated by epigenetic factors (Mateo-Bonmati et al., 2019); and the relative amounts of different forms of acetyl-CoA, which can be altered by environmental and metabolic factors. Together, these indicate that histone acetylation dynamics integrate metabolic activity to regulate plant responses to stress (Hu et al., 2019). Differential KEGG pathways were identified through joint bisulfite sequencing and transcriptome analysis (Figure 6C). There was an altered expression of key genes regulated by DNA methylation in these metabolic pathways (Figure 7). For example, genes involved in the isoquinoline alkaloid biosynthesis, the purine metabolism pathway, and the tropane, piperidine, and tyrosine metabolism pathway were upregulated. These results suggest that carbohydrate metabolism, carbon-nitrogen budget, and secondary metabolism are constitutively promoted in cold treatment. Genes related to amino acids and pyrimidine accumulation were upregulated in the cold shock treatment, potentially due to the differential accumulation of lysine and thymidine in both cold treatments.

To the knowledge of the authors, this is the first report on the regulation of lysine metabolic pathways by DNA methylation in plants, and the result of the present study shows different regulatory mechanisms for lysine degradation under different cold treatments (cold memory group and cold shock group). Lysine is an essential amino acid, and lysine level represents crop quality. Lysine is the classic target site of epigenetic modifications (histone methylation and acetylation; Zhang, 2008), and high accumulation of free lysine in endosperm induces multiple plant stress responses (Yang et al., 2018). Lysine metabolism is involved in the plant stress response in various ways (Yang et al., 2020a), and the results showed that lysine degradation is involved in the plant freezing response, with different lysine accumulation levels under various freezing treatments affected by DNA methylation (Figure 8B). Rutin is a flavonoid substance that is produced from phenylalanine as a precursor. Freezing treatment affects phenylalanine biosynthesis (Supplementary Table S2), with changes in methylation, but there was little difference between the two cold treatments (Figure 8B). To test the significance of DNA methylation for lysine and rutin enrichment, the DNA methylation inhibitor 5-aza-2'-deoxycytidine was applied during cold treatment, and a significant effect of DNA methylation on lysine content was observed. Most of the differentially expressed sequences were mapped to intergenic regions, which was likely caused by incomplete genome annotation. *De novo* sequencing should be carried out in the future to deeply analyze the genome of Tartary buckwheat varieties with excellent traits.

Plant memory involves multiple physiological, proteomic, transcriptional, and epigenetic changes, with the important role of epigenetic modification in plant memory confirmed by numerous studies (Iwasaki and Paszkowski, 2014; Kinoshita and Seki, 2014; Dean, 2017; Lamke and Baurle, 2017; He and Li, 2018; Turgut-Kara et al., 2020). In this study, the relationship between the effects of repeated environmental low-temperature stimulation (cold priming) and DNA methylation patterns, as well as changes in gene expression and metabolite enrichment after cold memory generation, was investigated, which leads to changes in some agronomic traits in crops (e.g., free lysine content). Future efforts to improve crops should utilize molecular module theory based on multiomics to improve important agronomic traits and increase tolerance to extreme ambient temperatures (Zhang et al., 2019). Here, differentially expressed genes, metabolites, and the possible roles of DNA methylation modification in cold priming in Tartary buckwheat were investigated. These findings provided comprehensive insights for the role of DNA methylation in cold priming (i.e., cold memory) and facilitated the breeding of ideal agronomic traits in Tartary buckwheat varieties.

## Data Availability Statement

The sequencing data generated in this study have been deposited in National Center for Biotechnology Information’s Gene Expression Omnibus and are accessible through the GEO Series accession nos. GSE138547, GSE138497 (BS-Seq), and GSE138546 (RNA-seq).

## Author Contributions

YS, ZJ, and LA contributed to conceive, design, and coordinate the experiments. YH, XM, LL, and XJ performed experiments. YS and ZJ analyzed the data. LA participated to the manuscript revision. YS wrote the manuscript. All authors contributed to the article and approved the submitted version.

### Conflict of Interest

The authors declare that the research was conducted in the absence of any commercial or financial relationships that could be construed as a potential conflict of interest.

## References

[ref1] Alonso C. Ramos-Cruz D. Becker C. (2019). The role of plant epigenetics in biotic interactions. New Phytol. 221, 731–737. 10.1111/nph.15408, PMID: 30156271 PMC6726468

[ref2] An N. Welch S. M. Markelz R. J. C. Baker R. L. Palmer C. M. Ta J. (2017). Quantifying time-series of leaf morphology using 2D and 3D photogrammetry methods for high-throughput plant phenotyping. Comput. Electron. Agric. 135, 222–232. 10.1016/j.compag.2017.02.001

[ref3] Ausin I. Greenberg M. V. C. Simanshu D. K. Hale C. J. Vashisht A. A. Simon S. A.. (2012). INVOLVED IN DE NOVO 2-containing complex involved in RNA-directed DNA methylation in Arabidopsis. Proc. Natl. Acad. Sci. U. S. A. 109, 8374–8381. 10.1073/pnas.1206638109, PMID: 22592791 PMC3365198

[ref4] Bruce T. J. A. Matthes M. C. Napier J. A. Pickett J. A. (2007). Stressful “memories” of plants: evidence and possible mechanisms. Plant Sci. 173, 603–608. 10.1016/j.plantsci.2007.09.002

[ref5] Butelli E. Licciardello C. Zhang Y. Liu J. Mackay S. Bailey P.. (2012). Retrotransposons control fruit-specific, cold-dependent accumulation of anthocyanins in blood oranges. Plant Cell 24, 1242–1255. 10.1105/tpc.111.095232, PMID: 22427337 PMC3336134

[ref6] Caldwell C. R. Britz S. J. Mirecki R. M. (2005). Effect of temperature, elevated carbon dioxide, and drought during seed development on the isoflavone content of dwarf soybean [*Glycine max* (L.) Merrill] grown in controlled environments. J. Agric. Food Chem. 53, 1125–1129. 10.1021/jf0355351, PMID: 15713029

[ref7] Cao Y. Tang X. F. Giovannoni J. Xiao F. M. Liu Y. S. (2012). Functional characterization of a tomato COBRA-like gene functioning in fruit development and ripening. BMC Plant Biol. 12:211. 10.1186/1471-2229-12-211, PMID: 23140186 PMC3533923

[ref8] Cerovic Z. G. Masdoumier G. Ben Ghozlen N. Latouche G. (2012). A new optical leaf-clip meter for simultaneous non-destructive assessment of leaf chlorophyll and epidermal flavonoids. Physiol. Plant. 146, 251–260. 10.1111/j.1399-3054.2012.01639.x, PMID: 22568678 PMC3666089

[ref9] Chang Y. N. Zhu C. Jiang J. Zhang H. M. Zhu J. K. Duan C. G. (2020). Epigenetic regulation in plant abiotic stress responses. J. Integr. Plant Biol. 62, 563–580. 10.1111/jipb.12901, PMID: 31872527

[ref10] Cheng J. Niu Q. Zhang B. Chen K. Yang R. Zhu J. K.. (2018). Downregulation of RdDM during strawberry fruit ripening. Genome Biol. 19:212. 10.1186/s13059-018-1587-x, PMID: 30514401 PMC6280534

[ref11] Chinnusamy V. Zhu J. K. (2009). Epigenetic regulation of stress responses in plants. Curr. Opin. Plant Biol. 12, 133–139. 10.1016/j.pbi.2008.12.006, PMID: 19179104 PMC3139470

[ref12] Christie P. J. Alfenito M. R. Walbot V. (1994). Impact of low-temperature stress on general phenylpropanoid and anthocyanin pathways - enhancement of transcript abundance and anthocyanin pigmentation in maize seedlings. Planta 194, 541–549. 10.1007/BF00714468

[ref13] Dean C. (2017). What holds epigenetic memory? Nat. Rev. Mol. Cell Biol. 18:140. 10.1038/nrm.2017.15, PMID: 28220047

[ref14] Ding Y. Fromm M. Avramova Z. (2012). Multiple exposures to drought 'train' transcriptional responses in Arabidopsis. Nat. Commun. 3:740. 10.1038/ncomms1732, PMID: 22415831

[ref15] Ding Y. L. Lv J. Shi Y. T. Gao J. P. Hua J. Song C. P.. (2019). EGR2 phosphatase regulates OST1 kinase activity and freezing tolerance in Arabidopsis. EMBO J. 38:e99819. 10.15252/embj.201899819, PMID: 30429206 PMC6315290

[ref16] D’Urso A. Brickner J. H. (2014). Mechanisms of epigenetic memory. Trends Genet. 30, 230–236. 10.1016/j.tig.2014.04.004, PMID: 24780085 PMC4072033

[ref17] Eckel M. Steinchen W. Batschauer A. (2018). ATP boosts lit state formation and activity of Arabidopsis cryptochrome 2. Plant J. 96, 389–403. 10.1111/tpj.14039, PMID: 30044014

[ref18] Fraudentali I. Ghuge S. A. Carucci A. Tavladoraki P. Angelini R. Cona A.. (2019). The copper amine oxidase AtCuAOdelta participates in abscisic acid-induced stomatal closure in Arabidopsis. Plants 8:183. 10.3390/plants8060183, PMID: 31226798 PMC6630932

[ref19] Gallusci P. Hodgman C. Teyssier E. Seymour G. B. (2016). DNA methylation and chromatin regulation during fleshy fruit development and ripening. Front. Plant Sci. 7:807. 10.3389/fpls.2016.00807, PMID: 27379113 PMC4905957

[ref20] Guo D. D. Juan J. X. Chang L. Y. Zhang J. J. Huang D. F. (2017). Discrimination of plant root zone water status in greenhouse production based on phenotyping and machine learning techniques. Sci. Rep. 7:8303. 10.1038/s41598-017-08235-z, PMID: 28811508 PMC5557858

[ref21] He Y. H. Li Z. C. (2018). Epigenetic environmental memories in plants: establishment, maintenance, and reprogramming. Trends Genet. 34, 856–866. 10.1016/j.tig.2018.07.006, PMID: 30144941

[ref22] He X. Li J. J. Chen Y. Yang J. Q. Chen X. Y. (2019). Genome-wide analysis of the WRKY gene family and its response to abiotic stress in buckwheat (*Fagopyrum tataricum*). Open Life Sci. 14, 80–96. 10.1515/biol-2019-0010 PMC7874777 33817140

[ref23] Hu Y. Lu Y. Zhao Y. Zhou D. X. (2019). Histone acetylation dynamics integrates metabolic activity to regulate plant response to stress. Front. Plant Sci. 10:1236. 10.3389/fpls.2019.01236, PMID: 31636650 PMC6788390

[ref24] Huang X. Y. Li M. Luo R. Zhao F. J. Salt D. E. (2019b). Epigenetic regulation of sulfur homeostasis in plants. J. Exp. Bot. 70, 4171–4182. 10.1093/jxb/erz218, PMID: 31087073

[ref25] Huang H. Liu R. Niu Q. Tang K. Zhang B. Zhang H.. (2019a). Global increase in DNA methylation during orange fruit development and ripening. Proc. Natl. Acad. Sci. U. S. A. 116, 1430–1436. 10.1073/pnas.1815441116, PMID: 30635417 PMC6347674

[ref26] Huff J. T. Zilberman D. (2012). Regulation of biological accuracy, precision, and memory by plant chromatin organization. Curr. Opin. Genet. Dev. 22, 132–138. 10.1016/j.gde.2012.01.007, PMID: 22336527

[ref27] Iwasaki M. Paszkowski J. (2014). Epigenetic memory in plants. EMBO J. 33, 1987–1998. 10.15252/embj.201488883, PMID: 25104823 PMC4195768

[ref28] Jung J. H. Park J. H. Lee S. To T. K. Kim J. M. Seki M.. (2013). The cold signaling attenuator HIGH EXPRESSION OF OSMOTICALLY RESPONSIVE GENE1 activates FLOWERING LOCUS C transcription via chromatin remodeling under short-term cold stress in Arabidopsis. Plant Cell 25, 4378–4390. 10.1105/tpc.113.118364, PMID: 24220632 PMC3875724

[ref29] Kinoshita T. Seki M. (2014). Epigenetic memory for stress response and adaptation in plants. Plant Cell Physiol. 55, 1859–1863. 10.1093/pcp/pcu125, PMID: 25298421

[ref30] Krueger F. Andrews S. R. (2011). Bismark: a flexible aligner and methylation caller for Bisulfite-Seq applications. Bioinformatics 27, 1571–1572. 10.1093/bioinformatics/btr167, PMID: 21493656 PMC3102221

[ref31] Lamke J. Baurle I. (2017). Epigenetic and chromatin-based mechanisms in environmental stress adaptation and stress memory in plants. Genome Biol. 18:124. 10.1186/s13059-017-1263-6, PMID: 28655328 PMC5488299

[ref32] Lang Z. Wang Y. Tang K. Tang D. Datsenka T. Cheng J.. (2017). Critical roles of DNA demethylation in the activation of ripening-induced genes and inhibition of ripening-repressed genes in tomato fruit. Proc. Natl. Acad. Sci. U. S. A. 114, E4511–E4519. 10.1073/pnas.1705233114, PMID: 28507144 PMC5465898

[ref33] Latzel V. Gonzalez A. P. R. Rosenthal J. (2016). Epigenetic memory as a basis for intelligent behavior in clonal plants. Front. Plant Sci. 7:1354. 10.3389/fpls.2016.01354, PMID: 27630664 PMC5006084

[ref34] Law J. A. Jacobsen S. E. (2010). Establishing, maintaining and modifying DNA methylation patterns in plants and animals. Nat. Rev. Genet. 11, 204–220. 10.1038/nrg2719, PMID: 20142834 PMC3034103

[ref35] Levy Y. (1980). Field determination of free proline accumulation and water-stress in lemon trees. HortScience 15, 302–303.

[ref36] Leyva A. Jarillo J. A. Salinas J. Martinez-Zapater J. M. (1995). Low temperature induces the accumulation of phenylalanine ammonia-lyase and chalcone synthase mRNAs of *Arabidopsis thaliana* in a light-dependent manner. Plant Physiol. 108, 39–46. 10.1104/pp.108.1.39, PMID: 12228452 PMC157303

[ref37] Li H. Yuan J. Wu M. Han Z. Li L. Jiang H.. (2018). Transcriptome and DNA methylome reveal insights into yield heterosis in the curds of broccoli (*Brassica oleracea* L var. italic). BMC Plant Biol. 18:168. 10.1186/s12870-018-1384-4, PMID: 30103674 PMC6090608

[ref38] Li C. Zhao H. Li M. Yao P. Li Q. Zhao X.. (2019). Validation of reference genes for gene expression studies in tartary buckwheat (*Fagopyrum tataricum* Gaertn.) using quantitative real-time PCR. PeerJ 7:e6522. 10.7717/peerj.6522, PMID: 30834187 PMC6396815

[ref39] Liu J. Hou H. Zhao L. Sun Z. Lu Y. Li H. (2019a). Mitigation of Cd accumulation in rice from Cd-contaminated paddy soil by foliar dressing of S and P. Sci. Total Environ. 690, 321–328. 10.1016/j.scitotenv.2019.06.332, PMID: 31299567

[ref40] Liu M. Y. Huang L. Ma Z. T. Sun W. J. Wu Q. Tang Z. Z.. (2019b). Genome-wide identification, expression analysis and functional study of the GRAS gene family in Tartary buckwheat (*Fagopyrum tataricum*). BMC Plant Biol. 19:342. 10.1186/s12870-019-1951-3, PMID: 31387526 PMC6683366

[ref41] Liu M. Y. Ma Z. T. Sun W. J. Huang L. Wu Q. Tang Z. Z.. (2019c). Genome-wide analysis of the NAC transcription factor family in Tartary buckwheat (*Fagopyrum tataricum*). BMC Genomics 20:113. 10.1186/s12864-019-5500-0, PMID: 30727951 PMC6366116

[ref42] Liu M. Y. Ma Z. T. Wang A. H. Zheng T. R. Huang L. Sun W. J.. (2018a). Genome-wide investigation of the auxin response factor gene family in Tartary buckwheat (*Fagopyrum tataricum*). Int. J. Mol. Sci. 19:3526. 10.3390/ijms19113526, PMID: 30423920 PMC6274889

[ref43] Liu M. Y. Ma Z. T. Zheng T. R. Sun W. J. Zhang Y. J. Jin W. Q.. (2018b). Insights into the correlation between physiological changes in and seed development of tartary buckwheat (*Fagopyrum tataricum* Gaertn.). BMC Genomics 19:648. 10.1186/s12864-018-5036-8, PMID: 30170551 PMC6119279

[ref44] Luo X. He Y. (2020). Experiencing winter for spring flowering: a molecular epigenetic perspective on vernalization. J. Integr. Plant Biol. 62, 104–117. 10.1111/jipb.12896, PMID: 31829495

[ref45] Mateo-Bonmati E. Casanova-Saez R. Ljung K. (2019). Epigenetic regulation of auxin homeostasis. Biomol. Ther. 9:623. 10.3390/biom9100623, PMID: 31635281 PMC6843323

[ref46] Molinier J. (2020). To be, or not to be, remethylated. Nat. Plants 6, 606–607. 10.1038/s41477-020-0696-1, PMID: 32514142

[ref47] Rea G. de Pinto M. C. Tavazza R. Biondi S. Gobbi V. Ferrante P.. (2004). Ectopic expression of maize polyamine oxidase and pea copper amine oxidase in the cell wall of tobacco plants. Plant Physiol. 134, 1414–1426. 10.1104/pp.103.036764, PMID: 15064377 PMC419818

[ref48] Rohde P. Hincha D. K. Heyer A. G. (2004). Heterosis in the freezing tolerance of crosses between two *Arabidopsis thaliana* accessions (Columbia-0 and C24) that show differences in non-acclimated and acclimated freezing tolerance. Plant J. 38, 790–799. 10.1111/j.1365-313X.2004.02080.x, PMID: 15144380

[ref49] Sahu P. P. Pandey G. Sharma N. Puranik S. Muthamilarasan M. Prasad M. (2013). Epigenetic mechanisms of plant stress responses and adaptation. Plant Cell Rep. 32, 1151–1159. 10.1007/s00299-013-1462-x, PMID: 23719757

[ref50] Song Y. Liu L. Feng Y. Wei Y. Yue X. He W.. (2015). Chilling- and freezing-induced alterations in cytosine methylation and its association with the cold tolerance of an alpine subnival plant, *Chorispora bungeana*. PLoS One 10:e0135485. 10.1371/journal.pone.0135485, PMID: 26270551 PMC4535906

[ref51] Song Y. Liu L. Ma X. (2019). CbADH1 improves plant cold tolerance. Plant Signal. Behav. 14:1612680. 10.1080/15592324.2019.1612680, PMID: 31056000 PMC6620001

[ref52] Thiebaut F. Hemerly A. S. Ferreira P. C. G. (2019). A role for epigenetic regulation in the adaptation and stress responses of non-model plants. Front. Plant Sci. 10:246. 10.3389/fpls.2019.00246, PMID: 30881369 PMC6405435

[ref53] Torrecillas A. Leon A. Delamor F. Ruizsanchez M. C. (1984). Determination of free proline levels in Citrus leaf-disks and its relation to xylem potential. Agrochimica 28, 371–378.

[ref54] Turgut-Kara N. Arikan B. Celik H. (2020). Epigenetic memory and priming in plants. Genetica 148, 47–54. 10.1007/s10709-020-00093-4, PMID: 32356021

[ref55] Wang J. W. Zhou X. Zhou Q. Cheng S. C. Wei B. D. Ji S. J. (2017). Low temperature conditioning alleviates peel browning by modulating energy and lipid metabolisms of 'Nanguo' pears during shelf life after cold storage. Postharvest Biol. Technol. 131, 10–15. 10.1016/j.postharvbio.2017.05.001

[ref56] Yang X. Sanchez R. Kundariya H. Maher T. Dopp I. Schwegel R.. (2020b). Segregation of an MSH1 RNAi transgene produces heritable non-genetic memory in association with methylome reprogramming. Nat. Commun. 11:2214. 10.1038/s41467-020-16036-8, PMID: 32371941 PMC7200659

[ref57] Yang Q. Q. Zhang C. Q. Chan M. L. Zhao D. S. Chen J. Z. Wang Q.. (2016). Biofortification of rice with the essential amino acid lysine: molecular characterization, nutritional evaluation, and field performance. J. Exp. Bot. 67, 4285–4296. 10.1093/jxb/erw209, PMID: 27252467 PMC5301931

[ref58] Yang Q. Q. Zhao D. S. Liu Q. Q. (2020a). Connections between amino acid metabolisms in plants: lysine as an example. Front. Plant Sci. 11:928. 10.3389/fpls.2020.00928, PMID: 32636870 PMC7317030

[ref59] Yang Q. Q. Zhao D. S. Zhang C. Q. Wu H. Y. Li Q. F. Gu M. H.. (2018). A connection between lysine and serotonin metabolism in eice endosperm. Plant Physiol. 176, 1965–1980. 10.1104/pp.17.01283, PMID: 29363563 PMC5841688

[ref60] Yao P. F. Deng R. Y. Huang Y. J. Stael S. Shi J. Q. Shi G. L.. (2019). Diverse biological effects of glycosyltransferase genes from Tartary buckwheat. BMC Plant Biol. 19:339. 10.1186/s12870-019-1955-z, PMID: 31382883 PMC6683379

[ref61] Zhang X. (2008). The epigenetic landscape of plants. Science 321, 489–492. 10.1126/science.1153996, PMID: 18436779

[ref62] Zhang J. Y. Li X. M. Lin H. X. Chong K. (2019). Crop improvement through temperature resilience. Annu. Rev. Plant Biol. 70, 753–780. 10.1146/annurev-arplant-050718-100016, PMID: 31035832

[ref63] Zhang L. Li X. Ma B. Gao Q. Du H. Han Y.. (2017). The Tartary buckwheat genome provides insights into rutin biosynthesis and abiotic stress tolerance. Mol. Plant 10, 1224–1237. 10.1016/j.molp.2017.08.013, PMID: 28866080

[ref64] Zhang Q. Li Y. Xu T. Srivastava A. K. Wang D. Zeng L.. (2016). The chromatin remodeler DDM1 promotes hybrid vigor by regulating salicylic acid metabolism. Cell Discov. 2:16027. 10.1038/celldisc.2016.27, PMID: 27551435 PMC4977722

[ref65] Zhang K. X. Logacheva M. D. Meng Y. Hu J. P. Wan D. P. Li L.. (2018). Jasmonate-responsive MYB factors spatially repress rutin biosynthesis in *Fagopyrum tataricum*. J. Exp. Bot. 69, 1955–1966. 10.1093/jxb/ery032, PMID: 29394372 PMC6018783

[ref66] Zhao Y. Antoniou-Kourounioti R. L. Calder G. Dean C. Howard M. (2020). Temperature-dependent growth contributes to long-term cold sensing. Nature 583, 825–829. 10.1038/s41586-020-2485-4, PMID: 32669706 PMC7116785

[ref67] Zhong S. Fei Z. Chen Y. R. Zheng Y. Huang M. Vrebalov J.. (2013). Single-base resolution methylomes of tomato fruit development reveal epigenome modifications associated with ripening. Nat. Biotechnol. 31, 154–159. 10.1038/nbt.2462, PMID: 23354102

[ref68] Zhou J. Applegate C. Alonso A. D. Reynolds D. Orford S. Mackiewicz M.. (2017a). Leaf-GP: an open and automated software application for measuring growth phenotypes for arabidopsis and wheat. Plant Methods 13:117. 10.1186/s13007-017-0266-3, PMID: 29299051 PMC5740932

[ref69] Zhou M. L. Sun Z. M. Ding M. Q. Logacheva M. D. Kreft I. Wang D.. (2017b). FtSAD2 and FtJAZ1 regulate activity of the FtMYB11 transcription repressor of the phenylpropanoid pathway in *Fagopyrum tataricum*. New Phytol. 216, 814–828. 10.1111/nph.14692, PMID: 28722263

[ref70] Zuther E. Schaarschmidt S. Fischer A. Erban A. Pagter M. Mubeen U.. (2019). Molecular signatures associated with increased freezing tolerance due to low temperature memory in Arabidopsis. Plant Cell Environ. 42, 854–873. 10.1111/pce.13502, PMID: 30548618

