# Supplementary material for: Roles of DNA Methylation in Cold Priming in Tartary Buckwheat
Source: Front Plant Sci. 2020 Dec 7;11:608540. doi: 10.3389/fpls.2020.608540 (PMC7750358; doi:10.3389/fpls.2020.608540)
Supplement: Supplementary file 8 [file Data_Sheet_1.docx]

***Journal of Integrative Plant Biology*** Supporting Information for

**Roles of DNA methylation in cold priming in Tartary buckwheat**

Yuan Song, Zhifeng Jia, Yukang Hou, Xiang Ma, Lizhen Li, Xing jin, Lizhe An

Yuan Song and Lizhe An

Email: songyuan@lzu.edu.cn and [lizhean@lzu.edu.cn](mailto:lizhean@lzu.edu.cn)

**This PDF file includes:**

Figs. S1 to S13

Supplementary text (Materials and Methods)

Supporting Information

Fig. S1 Field test of three Tartary buckwheat varieties from 2016 to 2018.

Fig. S2 Phenotypes of seeds of three Tartary buckwheat varieties within three days of germination.

Fig. S3 Phenotypic observation of three Tartary Buckwheat varieties in the three days before and after of seed germinations.

Fig. S4 Infrared and far infrared light determination of seeds.

Fig. S5 The morphologic observation through visible light of side and top surface test.

Fig. S6 Infrared and far infrared light determination of three-week-old seedlings in cold experiments.

Fig. S7 Different methylation (CG, CHG, and CHH) level of cytosine in in featured regions of the genome.

Fig.S8 Three-dimensional principal component analysis (PCA) on whole-genome bisulfite-sequencing samples.

Fig.S9 Histogram showing the numbers of DEGs in the cold memory and the cold shock relative to the control.

Fig.S10 GO analysis in the cold treatments relative to the control.

Fig.S11 KEGG analysis in the cold treatments relative to the control.

Fig.S12 Heatmap analysis of top 100 of differential expressed genes (DEGs) with differential methylation regions (DMRs).

Fig.S13 Clustering analysis of differentially expressed genes on chromosome 8 in S/C.

Table S1 Bisulfite sequencing coverage and conversion rate.

Table S2 Data of DNA methylation circus.

Table S3 Data of DNA methylation coverage.

Table S4 List of ChrFt8 genes comment regions.

Table S5 List of genes with coupling of the changes in DNA methylation and expression.

Table S6 List of significant down-regulation in the cold treatments relative to the control.

Table S7 Data of the two-dimensional scatter diagram of correlation analysis.


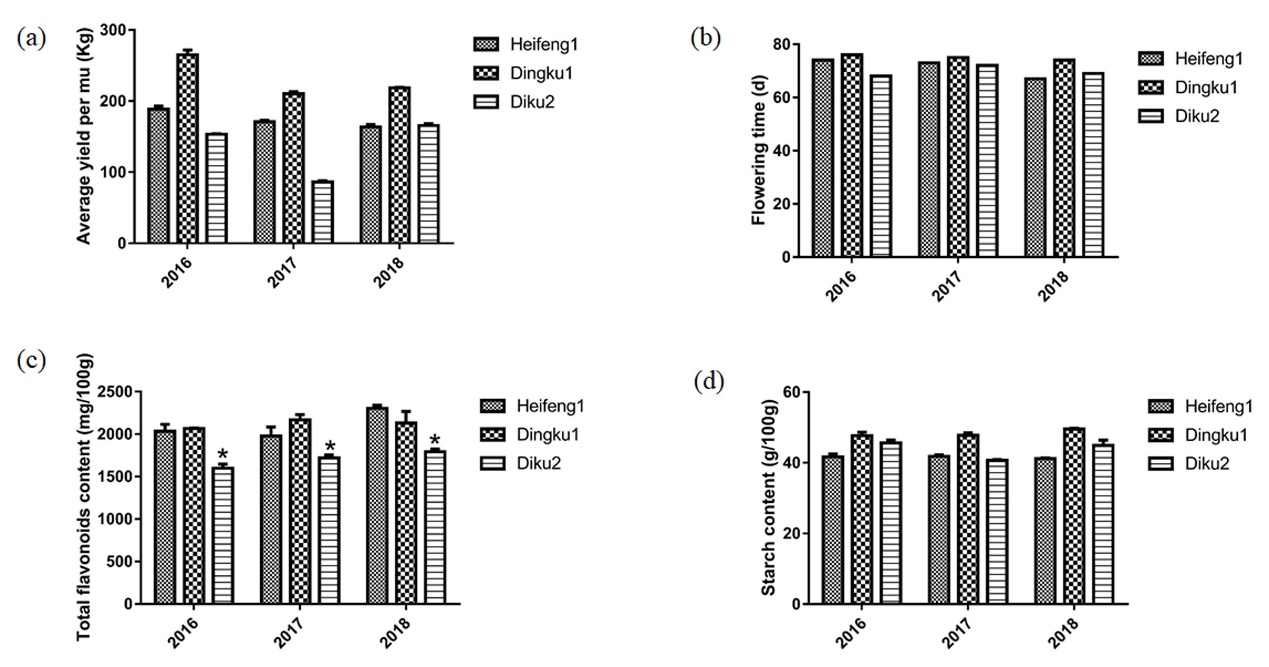


Fig. S1 Field test of three Tartary buckwheat varieties from 2016 to 2018. (a) The average yield per mu of three Tartary buckwheat varieties in the recent three years. (b) The record of flowering time of three Tartary buckwheat varieties in the recent three years. From the earliest one of the flowering started recording. (c), (d) Content of total flavonoids and starch in mature Seeds. The mean value was from more than 10 independent measurements, and error bars indicated ± SD.

(a)


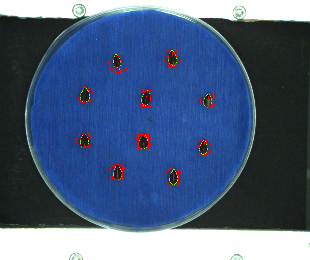

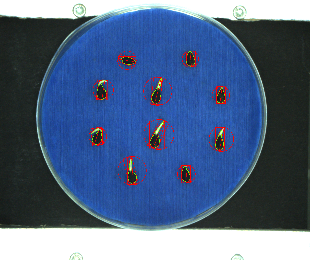

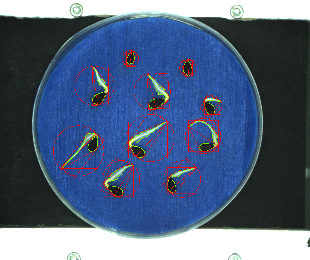


(b)


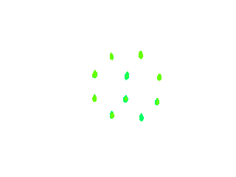

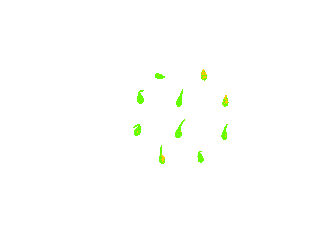

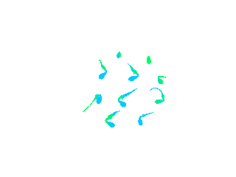


(c)


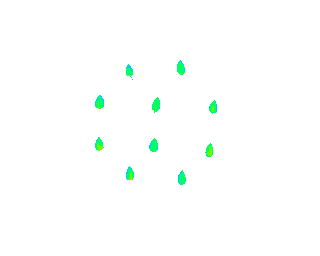

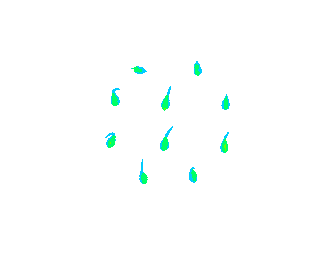

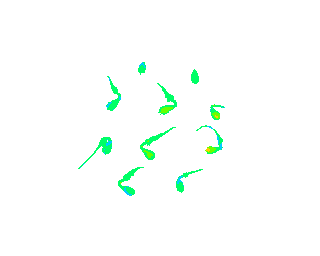


(d)


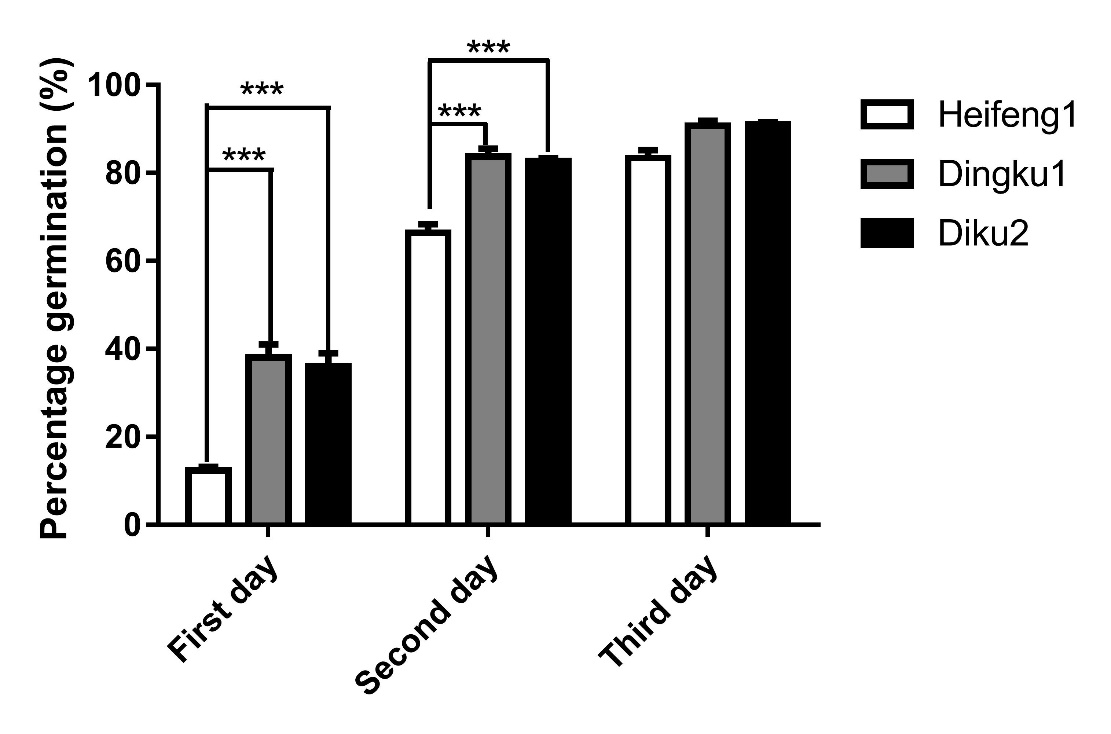


Fig. S2 Phenotypes of seeds of three Tartary buckwheat varieties within three days of germination. (a) Visible light---morphologic observation. (b) Infrared light--- relative humidity. (c) Near-infrared----relative water content. (d) Germination rate of three Tartary Buckwheat varieties. The mean value was from more than 30 independent measurements, and error bars indicated±SD. A total of 540 seeds were photographed and tested.


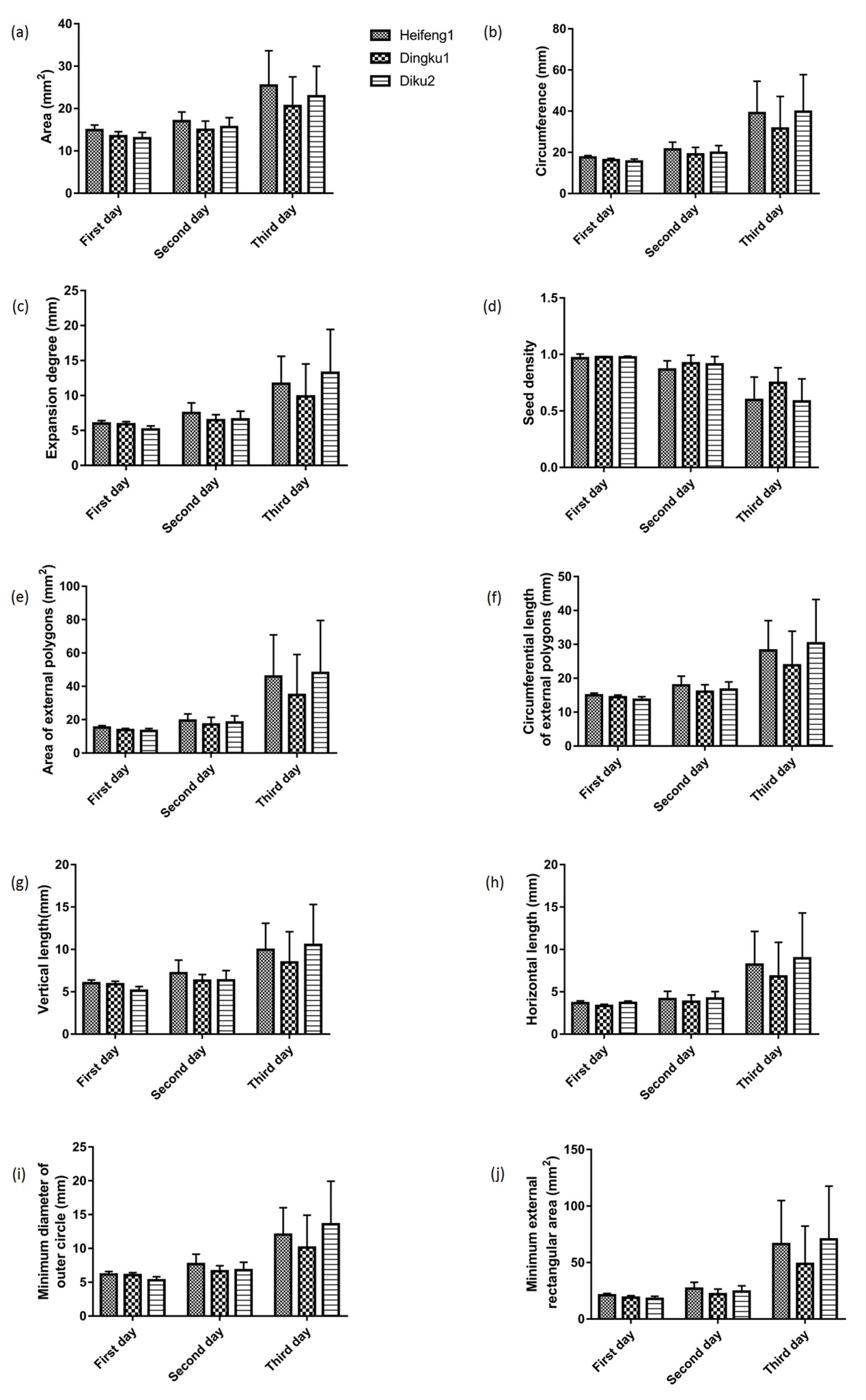


Fig. S3 Phenotypic observation of three Tartary Buckwheat varieties in the three days before and after of seed germinations. The mean value was from more than 30 independent measurements, and error bars indicated±SD.


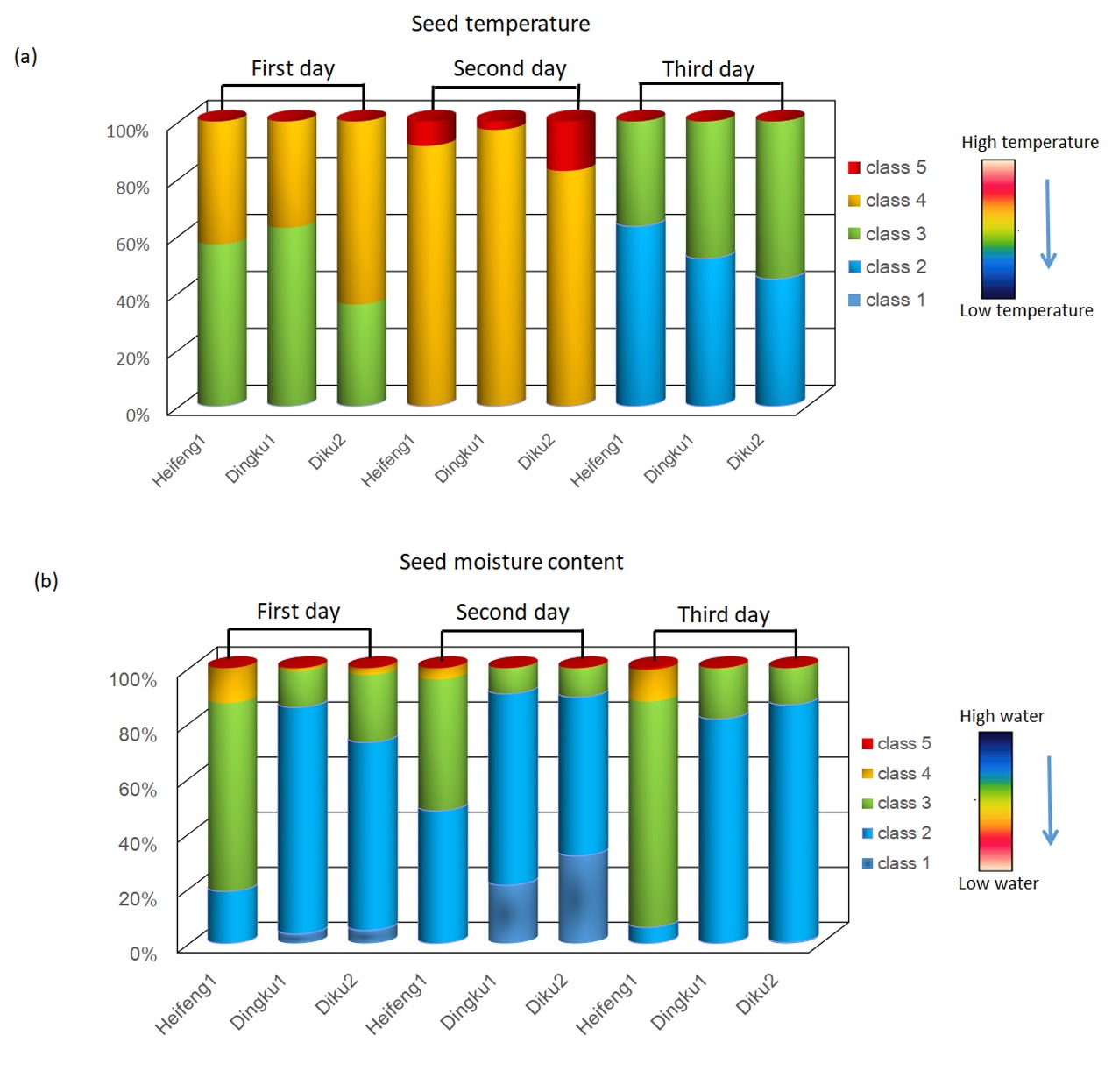
 Fig. S4 Infrared and far infrared light determination of seeds. (a) Seed temperature for three days of germination. Divided into five levels, the redder the color, the higher the temperature. (b) Seed moisture content for three days of germination. Divided into five levels, the bluer the color, the higher the moisture. The every mean value was from more than 30 independent measurements.


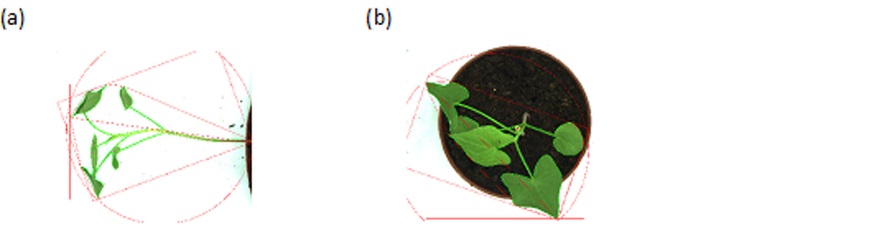


Fig. S5 (a)Visible light side test---morphologic observation. (b)Visible light top surface test---morphologic observation. Before and after cold treatment, each group of measurements was repeated 30 times (5 biological repeats and 6 technical repeats). A total of 270 seedlings were photographed and tested.

(a)


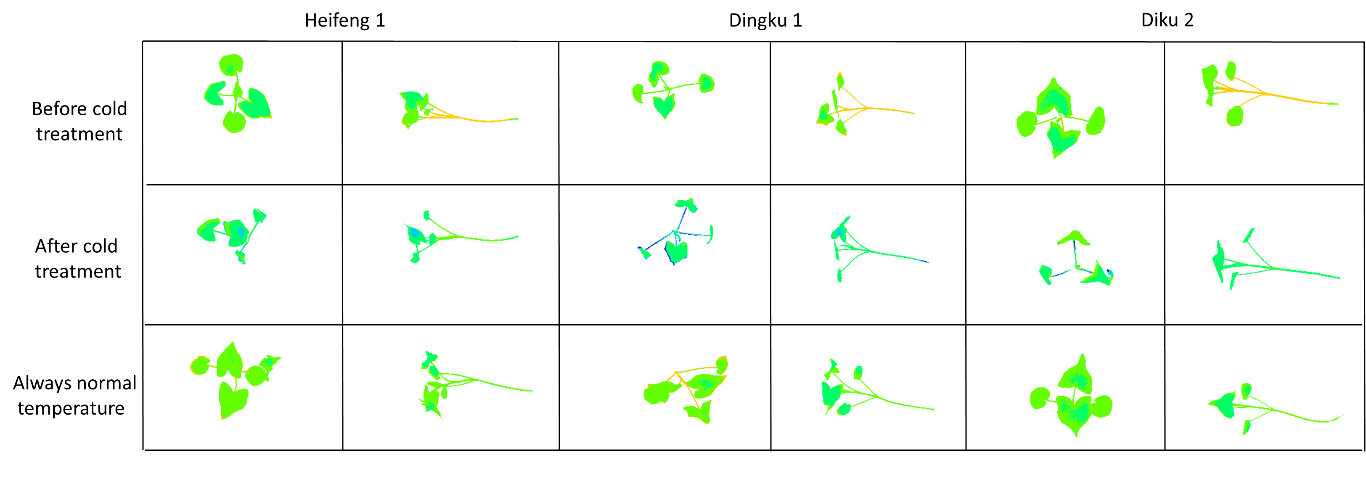


(b)


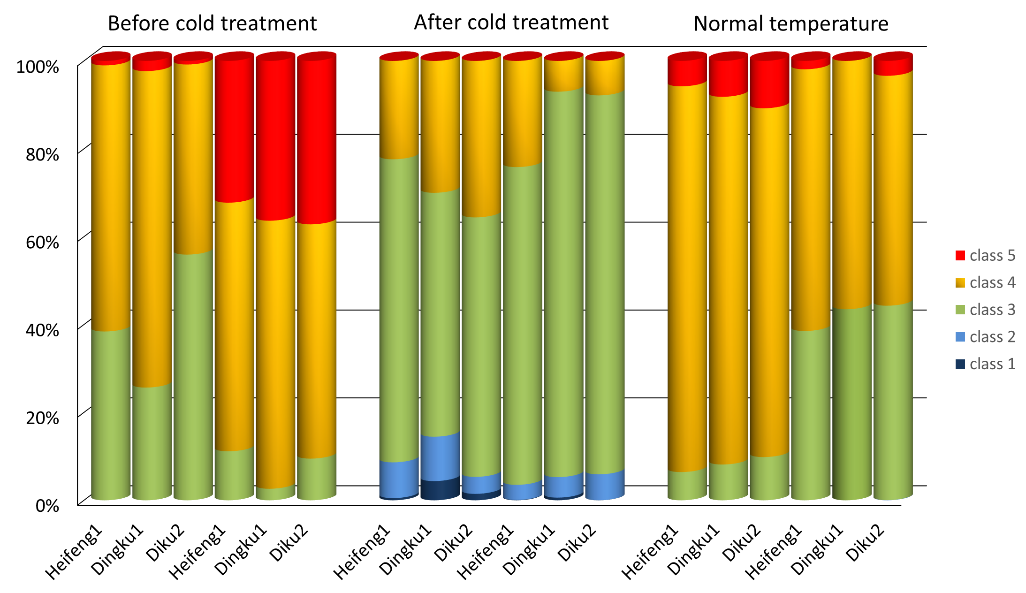


(c)


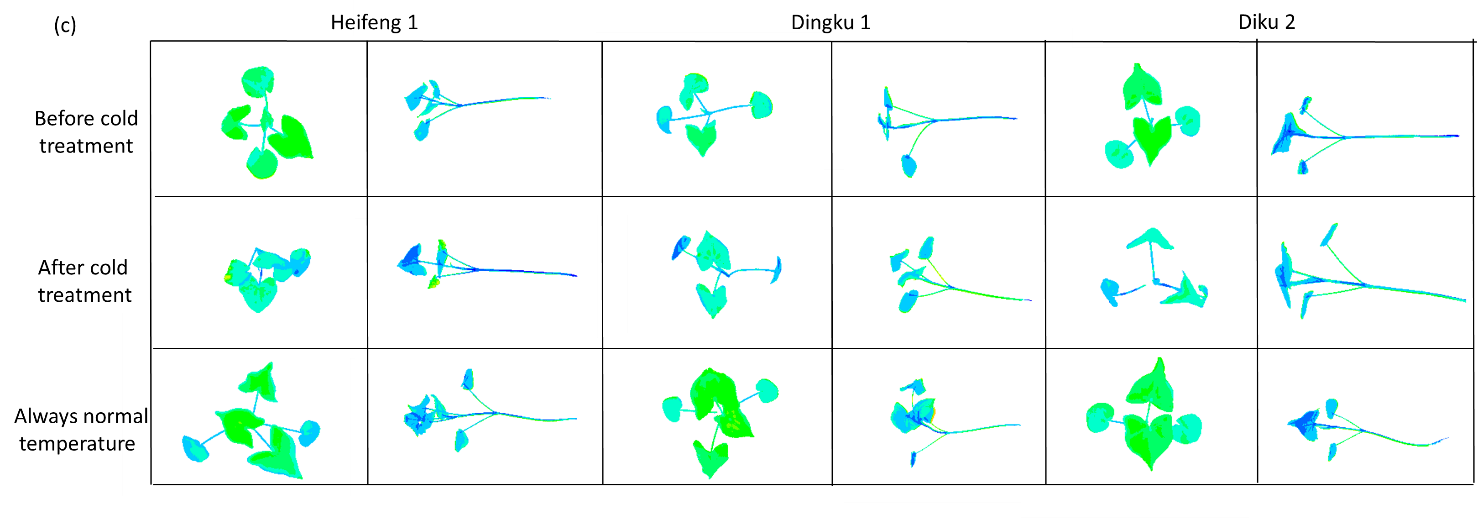


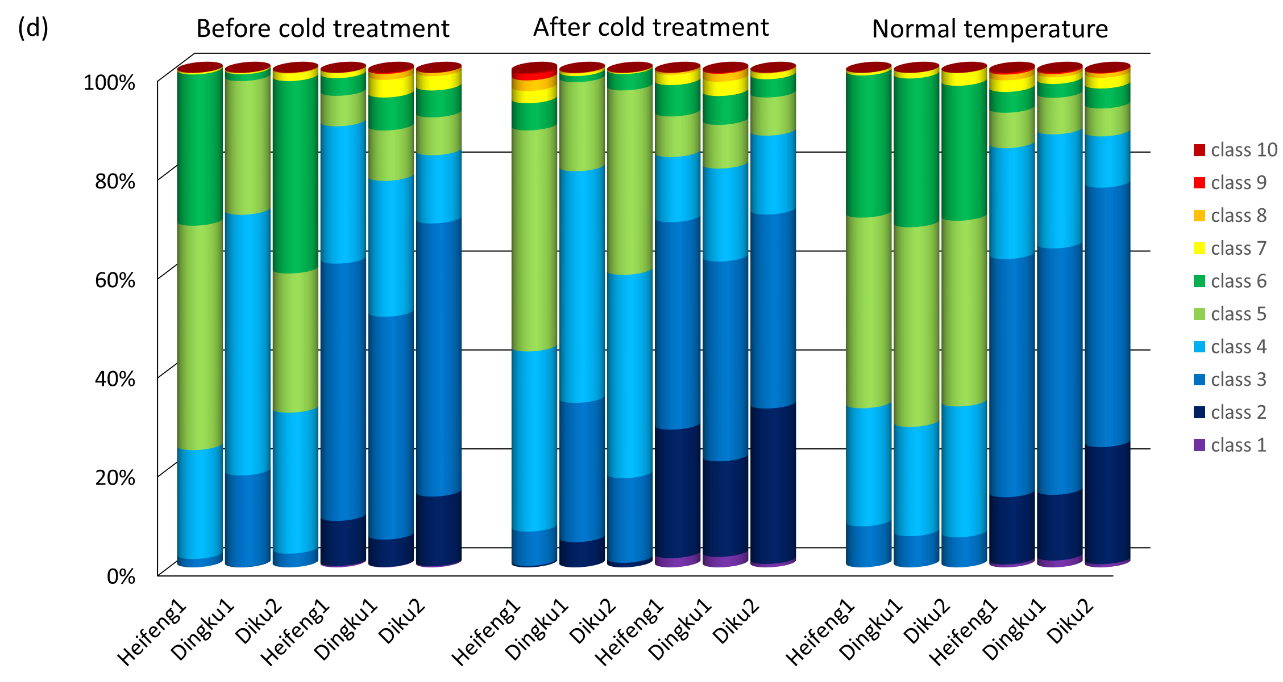


Fig. S6 Infrared and far infrared light determination of three-week-old seedlings in cold experiments. (a, b) Seedlings’ temperature before and after cold treatment. Divided into five levels, the redder the color, the higher the temperature. (c, d) Seedling’s moisture content before and after cold treatment. Divided into ten levels, the bluer the color, the higher the moisture. The more red and the drier. The every mean value was from more than 30 independent plant measurements. Take pictures from the top and side, respectively.


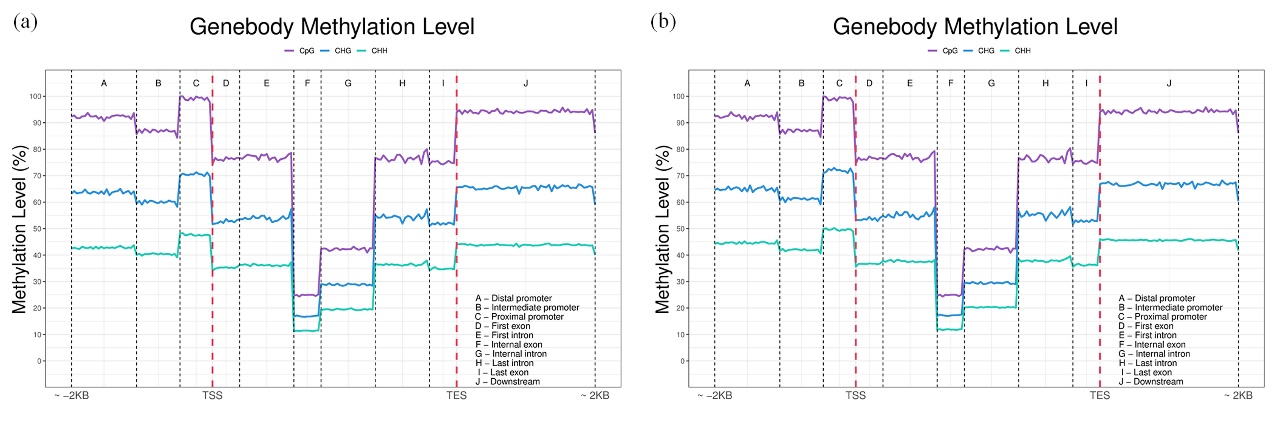


Fig. S7 Different methylation (CG, CHG, and CHH) level of cytosine in in featured regions of the genome. (a) Cold shock group; (b) Cold memory group.


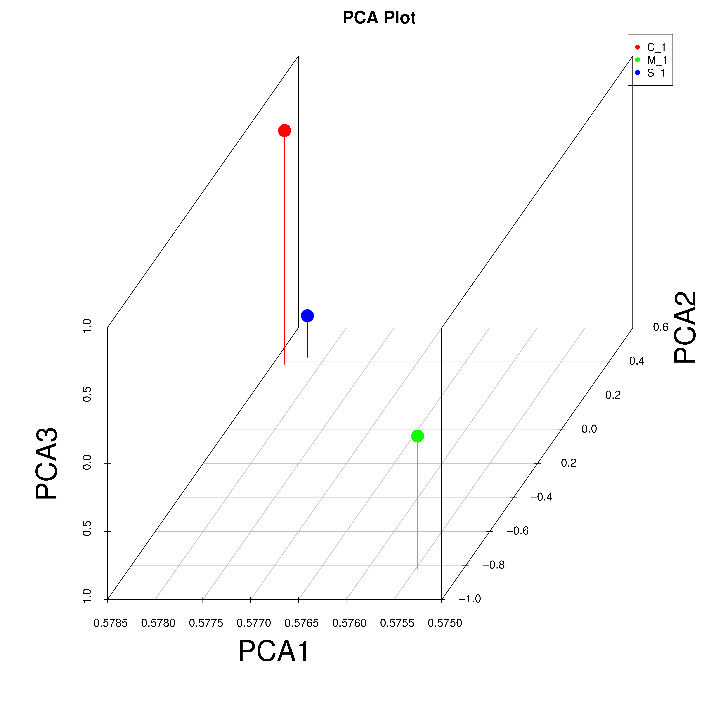


Fig.S8 Three-dimensional principal component analysis (PCA) showing good regional division among three different treatments of whole-genome bisulfite-sequencing samples in Dingku1 during cold stress.


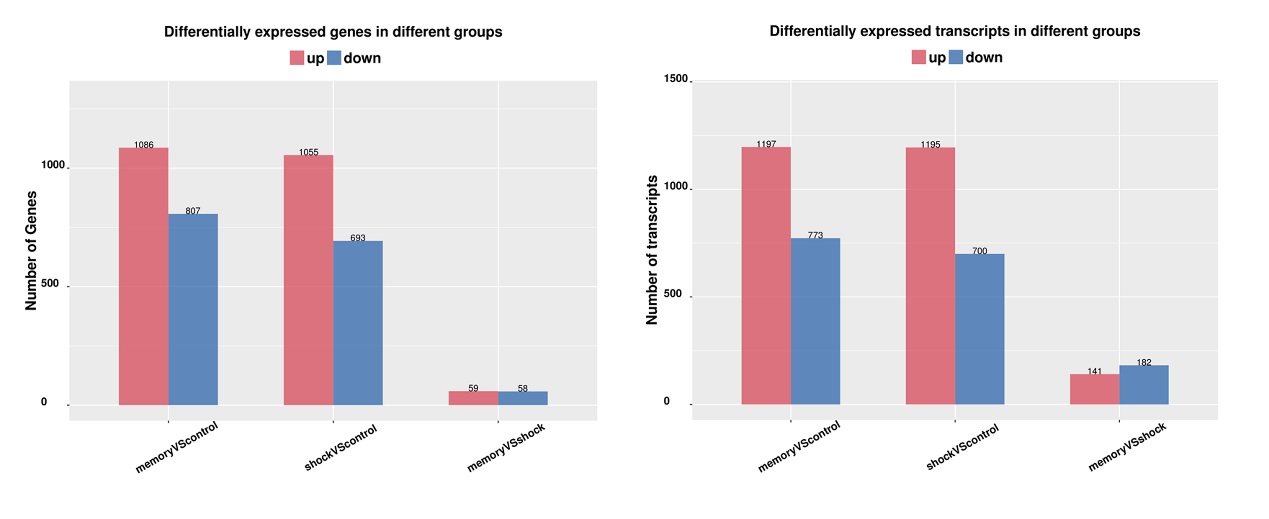


Fig.S9 Histogram showing the numbers of DEGs in the cold memory and the cold shock relative to the control.


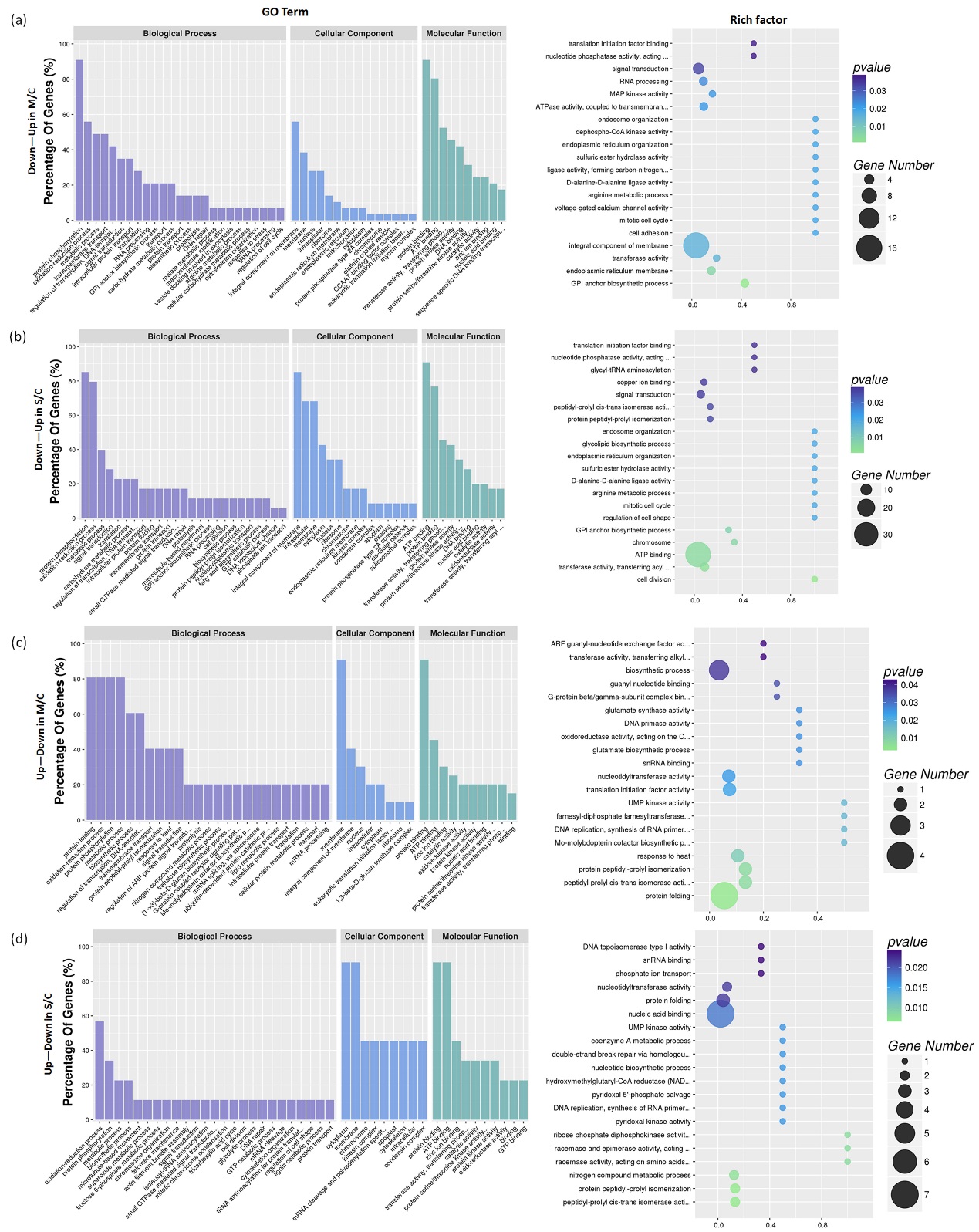


Fig.S10 Gene Ontology (GO) analysis in up-down (i.e. the down-regulated DEGs with hyper-DMR) and down-up (i.e. the up-regulated DEGs with hypo-DMR) in the cold treatments relative to the control (M/C and S/C).


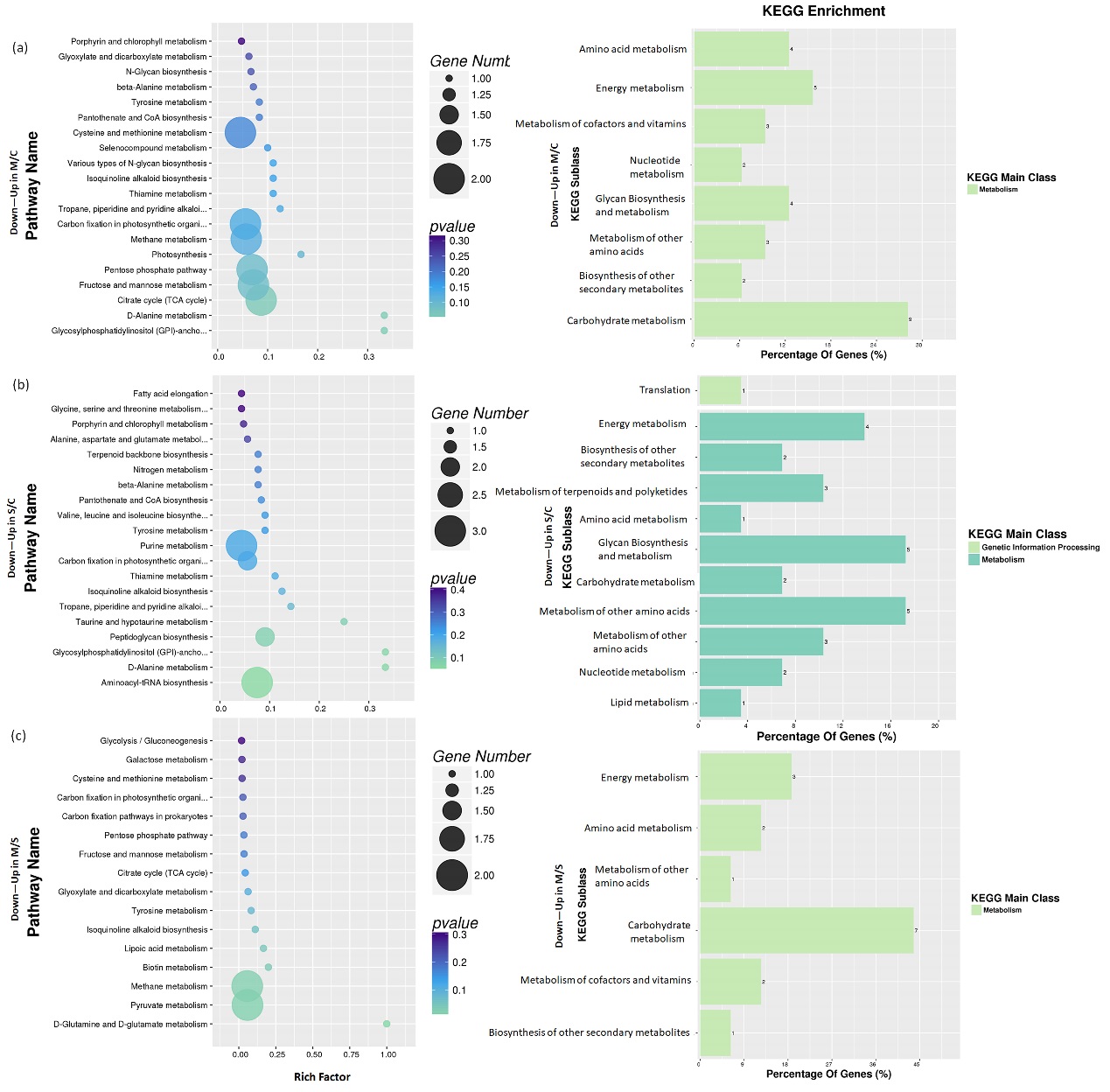


Fig.S11 Kyoto Encyclopedia of Genes and Genomes (KEGG) in down-up (i.e. the up-regulated DEGs with hypo-DMR) among the different treatments (M/C, S/C and M/S).


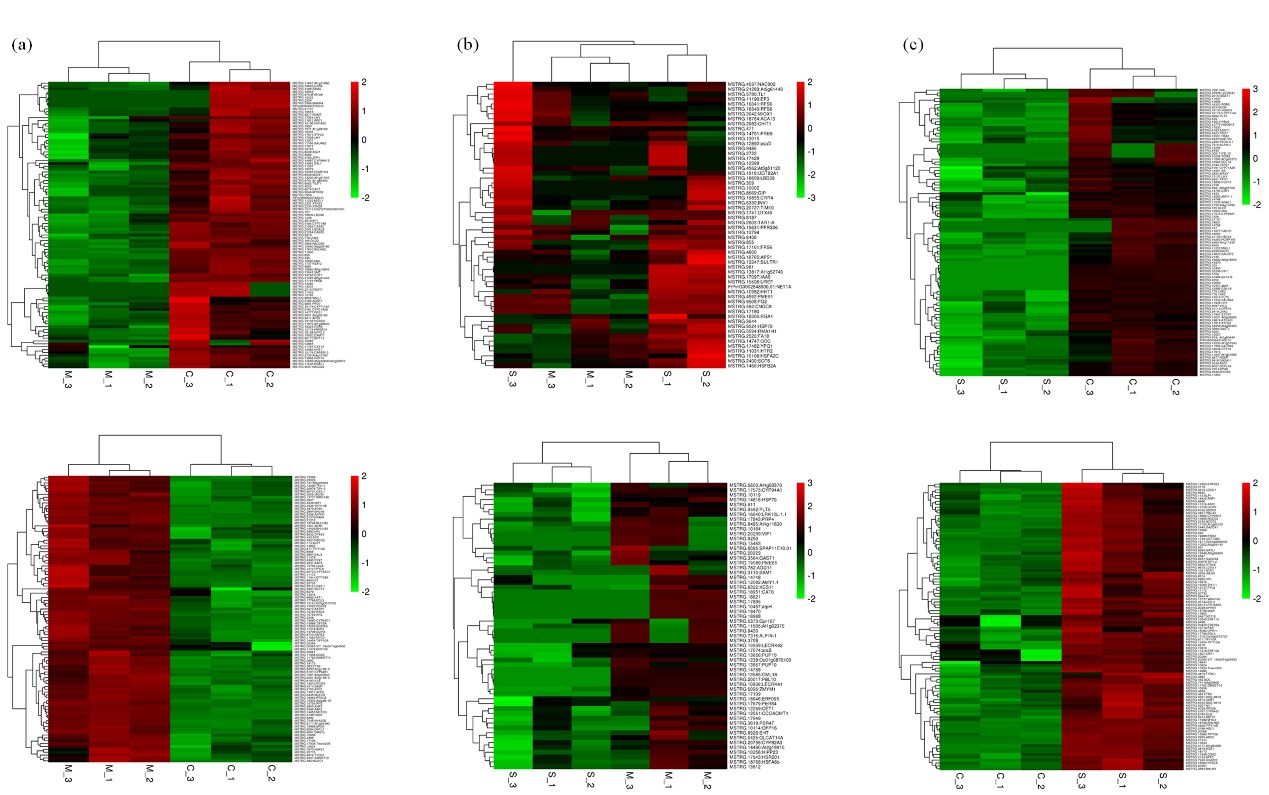


Fig.S12 Heatmap analysis of top 100 of differential expressed genes (DEGs) with differential methylation regions (DMRs).


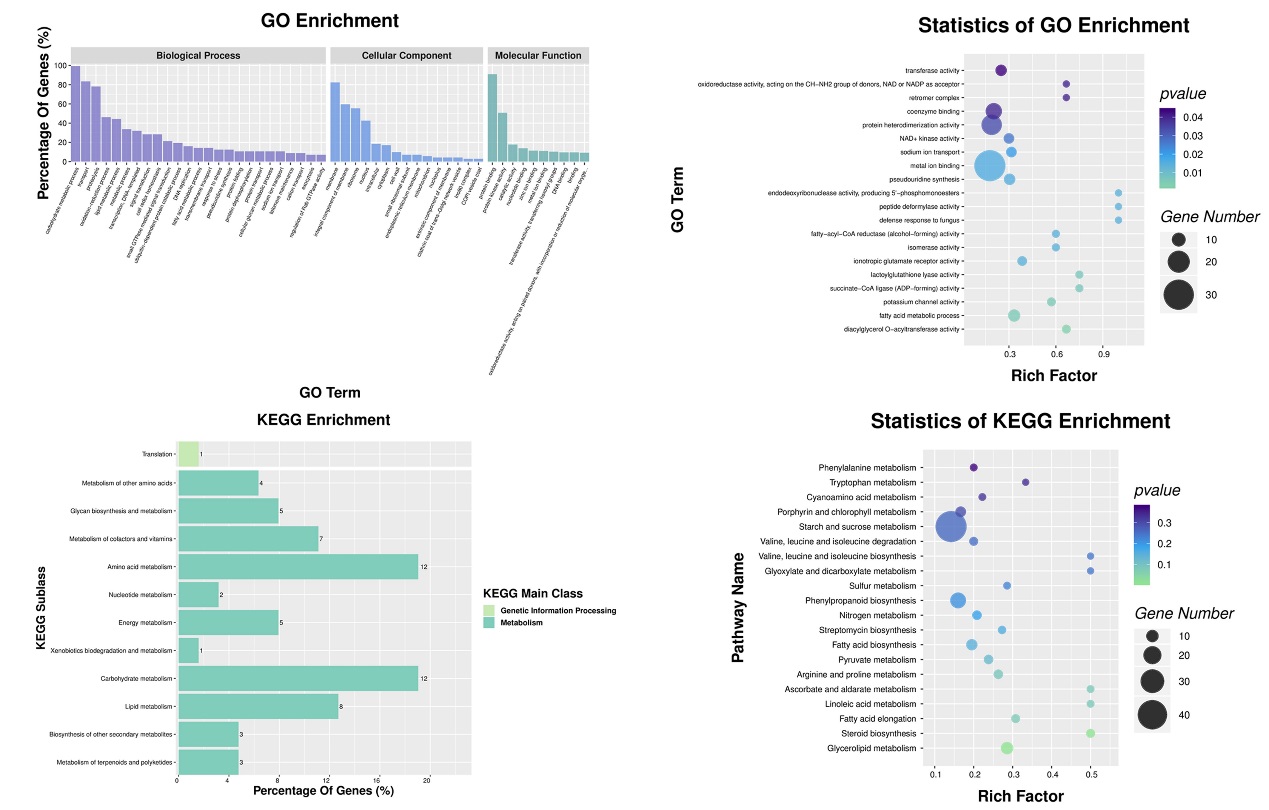


Fig.S13 GO and KEGG pathway of differentially expressed genes on chromosome 8 in S/C.

**Other supplementary materials for this manuscript include the following:**

**Supplementary Information Text**

**Materials and Methods**

**Library construction and sequencing**

Total DNA was extracted using QIAamp Fast DNA Tissue Kit (Qiagen, Dusseldorf, Germany) following the manufacturer's procedure. The quantity of DNA was measured by reading A260/280 ratios by spectrophotometer. When A260/280 ratios located range 1.8 to 2.0, DNA was available. The fragmented DNA samples by using sonication were subjected to bisulfite conversion. The Accel-NGS Methyl-Seq DNA Library Kit (Swift, MI, USA) was utilized for attaching adapters to single-stranded DNA fragments. Briefly, as protocol shown below, the *adaptase step* is a highly efficient, proprietary reaction that simultaneously performs end repair, tailing of 3’ ends, and ligation of the first truncated adapter complement to 3’ ends. *The Extension step* is used to incorporate truncated adapter 1 by a primer extension reaction. *The Ligation step* is used to add the second truncated adapter to the bottom strand only. *The Indexing PCR step* increases yield and incorporates full length adapters. Bead-based SPRI clean-ups are used to remove both oligonucleotides and small fragments, as well as to change enzymatic buffer composition. Finally, we performed the pair-end 2×150bp sequencing on an illumina Hiseq 4000 platform housed in the LC Sciences.

**Bioinformatics analysis**

Firstly, Cutadapt (Martin M, 2011) and perl scripts in house were used to remove the reads that contained adapter contamination, low quality bases and undetermined bases. Then sequence quality was verified using FastQC (http://www.bioinformatics.babraham.ac.uk/projects/fastqc/). Reads that passed quality control were mapped to reference genome using WALT (Chen, H., 2016). After alignment, reads were further deduplicated using samtool (Li, H., et al. 2009). For each cytosine site (or guanine corresponding to a cytosine on the opposite strand) in the reference genome sequence, the DNA methylation level was determined by the ratio of the number of reads supporting C (methylated) to that of total reads (methylated and unmethylated) using per scripts in house and MethPipe (Song, Q., et al. 2013). Differentially methylated regions (DMRs) were calculated by R package-MethylKit (Akalin, A., et al. 2012) with default parameters (1000 bp slide windows, 500 bp overlap, p value < 0.05).

**RNA-seq reads mapping:**

we aligned reads of sample A and sample B to the UCSC (http://genome.ucsc.edu/) homo sapiens reference genome using HISAT package, which initially remove a portion of the reads based on quality information accompanying each read and then maps the reads to the reference genome. HISAT allows multiple alignments pe read (up to 20 by default) and a maximum of two mismatchs when mapping the reads to the reference. HISAT build a database of potential splice junctions and confirms these by comparing the previously unmapped reads against the database of putative junctions.

**Transcript abundance estimation and differentially expressed testing:**

The mapped reads of each sample were assembled using StringTie. Then, all transcriptomes from Samples were merged to reconstruct a comprehensive transcriptome using perl scripts. After the ﬁnal transcriptome was generated, StringTie and edgeR was used to estimate the expression levels of all transcripts. StringTie was used to perform expression level for mRNAs by calculating FPKM. The differentially expressed mRNAs and genes were selected with log2 (fold change) >1 or log2 (fold change) <-1 and with statistical significance (p value < 0.05) by R package.

All metabolic and non-metabolic pathways were acquired from the KEGG pathways database (http://www.kegg.jp/kegg/pathway.html), and the KEGG pathways clustering analysis of differentially expressed genes was achieved via expression analysis systematic explorer (EASE).

The GO accessions were mapped to GO terms according to molecular function, biological process, and cellular component ontologies (http://www.geneontology.org/), and were performed with the GSEABase package of BioConductor (http:// www.bioconductor.org/). GO enrichment was statistically tested using the hypergeometric test and the differentially expressed genes from biological processes were clustered. The Bonferroni correction method was used for multiple test correction.

**Liquid phase parameter description:**

All samples were acquired by the LC-MS system followed machine orders. Firstly, all chromatographic separations were performed using an ultra performance liquid chromatography (UPLC) system (SCIEX, UK). An ACQUITY UPLC T3 column (100mm*2.1mm, 1.8µm, Waters, UK) was used for the reversed phase separation. The column oven was maintained at 35°C. The flow rate was 0.4 ml/min and the mobile phase consisted of solvent A (water, 0.1% formic acid) and solvent B (Acetonitrile, 0.1% formic acid). Gradient elution conditions were set as follows: 0～0.5 min, 5% B; 0.5～7 min, 5% to 100% B; 7~8 min, 100% B; 8～8.1 min, 100% to 5% B; 8.1～10 min, 5%B. The injection volume for each sample was 4 µl.

**Mass spectrometry parameter description:**

A high-resolution tandem mass spectrometer TripleTOF5600plus (SCIEX, UK) was used to detect metabolites eluted form the column. The Q-TOF was operated in both positive and negative ion modes. The curtain gas was set 30 PSI, Ion source gas1 was set 60 PSI, Ion source gas2 was set 60 PSI, and an interface heater temperature was 650 ℃.For positive ion mode, the Ionspray voltage floating were set at 5000 V, respectively. For negative ion mode, the Ionspray voltage floating were set at -4500V, respectively. The mass spectrometry data were acquired in IDA mode. The TOF mass range was from 60 to 1200 Da. The survey scans were acquired in 150 ms and as many as 12 product ion scans were collected if exceeding a threshold of 100 counts per second (counts/s) and with a 1+ charge-state. Total cycle time was fixed to 0.56 s.Four time bins were summed for each scan at a pulser frequency value of 11 kHz through monitoring of the 40 GHz multichannel TDC detector with four-anode/channel detection. Dynamic exclusion was set for 4 s. During the acquisition, the mass accuracy was calibrated every 20 samples. Furthermore, in order to evaluate the stability of the LC-MS during the whole acquisition, a quality control sample (Pool of all samples) was acquired after every 10 samples.

**Information analysis description：**

The acquired MS data pretreatments including peak picking, peak grouping, retention time correction, second peak grouping, and annotation of isotopes and adducts was performed using XCMS software (Smith *et al.*, 2006). LC−MS raw data files were converted into mzXML format and then processed by the XCMS, CAMERA and metaX toolbox implemented with the R software. Each ion was identified by combining retention time (RT) and m/z data. Intensities of each peaks were recorded and a three dimensional matrix containing arbitrarily assigned peak indices (retention time-m/z pairs), sample names (observations) and ion intensity information (variables) was generated.

The online KEGG, HMDB database was used to annotate the metabolites by matching the exact molecular mass data (m/z) of samples with those from database. If a mass difference between observed and the database value was less than 10 ppm, the metabolite would be annotated and the molecular formula of metabolites would further be identified and validated by the isotopic distribution measurements. We also used a in-house fragment spectrum library of metabolites to validate the metabolite identification.

The intensity of peak data was further preprocessed by metaX (Wen *et al.*, 2017). Those features that were detected in less than 50% of QC samples or 80% of biological samples were removed, the remaining peaks with missing values were imputed with the k-nearest neighbor algorithm to further improve the data quality. PCA was performed for outlier detection and batch effects evaluation using the pre-processed dataset. Quality control-based robust LOESS signal correction was fitted to the QC data with respect to the order of injection to minimize signal intensity drift over time. In addition, the relative standard deviations of the metabolic features were calculated across all QC samples, and those > 30% were then removed.

Student t-tests were conducted to detect differences in metabolite concentrations between 2 phenotype. The *P* value was adjusted for multiple tests using an FDR (Benjamini–Hochberg). Supervised PLS-DA was conducted through metaX to discriminate the different variables between groups. The VIP value was calculated. A VIP cut-off value of 1.0 was used to select important features.
